# An integrated model of school students’ academic achievement and life satisfaction. Linking soft skills, extracurricular activities, self-regulated learning, motivation, and emotions

**DOI:** 10.1007/s10212-022-00601-4

**Published:** 2022-01-28

**Authors:** Tommaso Feraco, Dario Resnati, Davide Fregonese, Andrea Spoto, Chiara Meneghetti

**Affiliations:** 1 Department of General Psychology, University of Padova, via Venezia 8, Padua, Italy; 2 Pentathlon Srl, Napoli, Italy

**Keywords:** Academic achievement, Soft skills, Motivation, Self-regulated learning, Achievement emotions, Extracurricular activities, Life satisfaction

## Abstract

The role of soft skills at school is still debated, but they have emerged as important factors for students’ academic achievement and life satisfaction. This study focuses on the combined influence of soft skills (in terms of adaptability, curiosity, leadership, initiative, perseverance, and social awareness), extracurricular activities, achievement emotions, self-regulated learning, motivation, and cognitive abilities on academic achievement and life satisfaction. A sample of 603 students (5th to 12th graders) participated in the study. The results of a Bayesian path analysis based on meta-analytical priors show that soft skills were (i) directly positively associated with students’ achievement emotions, self-regulated learning, motivation, and life satisfaction and (ii) indirectly related with academic achievement through the mediation of self-regulated learning and motivation. On the other hand, only soft skills and achievement emotions were directly related to life satisfaction. Extracurricular activities showed a positive association with both soft skills and cognitive abilities. These results are the first to demonstrate the importance of soft skills and extracurricular activities when integrating all the above-mentioned factors in a model of students’ academic achievement and life satisfaction.

**Supplementary Information:**

The online version contains supplementary material available at 10.1007/s10212-022-00601-4.

## Introduction

Soft skills are internationally recognized as important personal characteristics that promote an individual’s wellbeing and success (e.g., European Commission, 2016; Heckman, 2011; the Italian Ministry of Education, University and Research [MIUR], 2018; the National Research Council, Pellegrino & Hilton, 2012). Their role in educational settings is still debated, however, and they have not been clearly integrated in other learning models (Feraco et al., 2021). In this study, we select six soft skills based on the World Economic Forum model (WEF, 2016) to study how—together with achievement emotions (Pekrun et al., 2007), extracurricular activities (ECA, Shulruf, 2010), self-regulated learning (SRL, Panadero, 2017), motivation, and cognitive abilities (Roth et al., 2015)—they influence academic achievement and wellbeing, in terms of life satisfaction (i.e., one’s cognitive evaluation of his/her contentment with life; Diener et al., 1985). Considering life satisfaction gives us a more holistic picture of the students, envisaging them as more than just learners and allowing to study and promote their wellbeing (Suldo et al., 2006; Weber et al., 2016). Indeed, even if academic achievement is a widely used indicator of scholastic performance that proved crucial to people’s careers (Spinath, 2012), it has revealed only a weak association with students’ life satisfaction (Ng et al., 2015; Suldo et al., 2006). In other words, considering academic achievement and life satisfaction together might help us see how soft skills, learning-related variables, and activities work together to favor them, thus providing useful insight on what factors educators, school psychologists, and policymakers should bear in mind.

The following sections will define SRL, academic motivation, achievement emotions, and cognitive abilities to specify the working model. Soft skills and ECA will subsequently be integrated into this model.

### Self-regulated learning, motivation, achievement emotions, and cognition

SRL, motivation, and cognitive abilities are three of the main and most often studied predictors of academic achievement (Huang, 2012; Richardson et al., 2012; Roth et al., 2015; Winne & Nesbit, 2010).

Cognitive ability is the strongest direct predictor of academic achievement (Roth et al., 2015). It includes the skills involved in thinking, memorizing, and processing information from study materials, while it is generally unrelated or only slightly related with SRL, motivation, or life satisfaction (Köller et al., 2019; Kriegbaum et al., 2018; Lavrijsen et al., 2021; Suldo et al., 2006; Zuffianò et al., 2013).

SRL is a broad construct that covers cognitive, behavioral, and affective aspects of learning (Panadero, 2017) focusing on the active role of students, who self-regulate their learning process in a recursive cycle thought to involve three main phases: preparatory, performance, and reappraisal (Efklides, 2011; Pintrich, 2004; Winne & Hadwin, 1998; Zimmerman, 2008). In other words, self-regulated learners set themselves functional goals and organize their studies; know and use study strategies; and have a metacognitive awareness of their learning process (Panadero, 2017). More recently, models of SRL (Ben-Eliyahu, 2019; Efklides, 2011) have placed emphasis on the impact of affective and motivational factors, which have been seen as a direct part of SRL, or as influencing SRL processes (Panadero, 2017).

Motivation is again a broad construct indicating an individual’s internal drive to achieve a certain goal, and many models of academic motivation have been proposed (Kriegbaum et al., 2018; Pintrich, 2003). Considering the different components of academic motivation, for the purposes of this study, we conceptualize it in terms of self-efficacy (Bandura, 1997), theories of intelligence (Dweck, 1999), and learning goals (Dweck & Leggett, 1988). All three factors have emerged as positive predictors of academic achievement (Costa & Faria, 2018; Richardson et al., 2012). They respectively represent the sense of being able to succeed in academic tasks, the belief that intelligence is malleable, and the focus on gaining competences and knowledge when studying. These concepts are related to each other, and to SRL (Burnette et al., 2013; Efklides, 2011; Huang, 2012; Richardson et al., 2012; Sitzmann & Ely, 2011), and they can be considered a single factor representing students’ motivation (Mega et al., 2014).

SRL and motivation could both have a role in life satisfaction too. SRL might promote the choice of a pathway towards wellbeing (Panadero, 2017), and motivation may foster life satisfaction through an individual’s self-determination (Peters et al., 2018). Some researchers have found, for example, that learning goals and self-efficacy are generally associated with life satisfaction (Antaramian, 2017; Danielsen et al., 2009; Diseth et al., 2012; Suldo et al., 2006). The importance of SRL and motivation to students’ academic achievement has led, however, to less attention being paid to their influence on students’ wellbeing, which might be limited to scholastic satisfaction, rather than general life satisfaction. This issue is worth investigating further.

Another affective aspect of learning that has recently attracted attention concerns the emotions that relate directly to learning activities and outcomes: achievement emotions (Pekrun, 2006). They are an important aspect of students’ school life because they affect their motivation, SRL, and academic achievement (Mega et al., 2014; Pekrun et al., 2002, 2007). According to the control-value theory (Pekrun, 2006; Pekrun et al., 2007), positive achievement emotions should promote metacognitive thoughts, the use of creative learning strategies, and a stronger motivation to study, for instance. Conversely, negative emotions would be detrimental since they would induce students to adopt rigid, ineffective strategies, and lower their motivation and interest in their study materials. Achievement emotions are therefore thought to influence academic achievement both directly and through the mediation of SRL and motivation (Daniels et al., 2009; Heffner & Antaramian, 2016; Huang, 2011; Linnenbrink, 2007; Mega et al., 2014; Pekrun et al., 2002; Pekrun et al., 2011; Putwain et al., 2020). In line with this evidence and with the model proposed by Mega et al. (2014), for the purposes of the present study, we will categorize emotions depending on their valence (positive-negative) and consider achievement emotions as direct predictors of SRL and motivation and as potential direct predictors of academic achievement. Achievement emotions also relate to a student’s wellbeing, however. Emotions generally have a strong association with life satisfaction (Diener, 2012; Heffner & Antaramian, 2016), and this has been confirmed for achievement emotions too (Hagenauer et al., 2018; Heffner & Antaramian, 2016; Karatzias et al., 2002; King & dela Rosa, 2019).

Following recent suggestions, all the factors driving the individual’s trajectory through development should be considered together as part of the intraindividual model (Ben-Eliyahu (2019); Ben-Eliyahu & Bernacki, 2015). Among these factors, which include cognitive abilities, SRL, motivation, and emotions, the authors included personal skills—what we now aim to study in terms of soft skills.

### Soft skills

Soft skills are malleable personal qualities that regulate emotions, behavior, and cognition, enabling us to achieve our goals (Park et al., 2004; Robles, 2012). Soft skills are of primary importance to labor market outcomes, favoring individuals’ chances of employment, success in the workplace, and creative outputs (e.g., Deming, 2017; Heckman & Kautz, 2012; WEF, 2016). This has led international institutions to focus on them, and how to nurture them (European Commission, 2016; Pellegrino & Hilton, 2012) from school age onwards (Heckman, 2011; MIUR, 2018). Their role in education has yet to be systematically explored, however, and the numerous soft skill frameworks proposed in the literature are somewhat confusing (see Bhagra & Sharma, 2018). Among the plethora of models, the WEF has developed a tripartite framework of soft skills based on educational and economic needs for the twenty-first century (WEF, 2016). This model has important practical implications, it is succinct, and it was recently applied to academic achievement (Feraco et al., 2021). Here, we limit our study to the six soft skills of the “qualities” branch of the WEF model: adaptability (the capacity to constructively regulate psycho-behavioral functioning in response to new, or uncertain circumstances; Martin et al., 2012); curiosity (the desire to learn and discover that drives people to explore and acquire new information; Berlyne, 1960); initiative (the tendency to intentionally improve oneself; Robitschek et al., 2012); leadership (the ability to help and influence others to reach team success; Peterson & Seligman, 2004); perseverance (the inclination to work hard even when facing difficulties; Duckworth et al., 2007); and social awareness (the sense of responsibility towards the community; Peterson & Seligman, 2004).

### Soft skills, academic achievement, and life satisfaction

As mentioned earlier, the literature on the relation between soft skills and academic achievement is poorly structured, and previous studies approached the various soft skills as individual constructs without considering what they may have in common as part of soft skills. According to the sizable body of reported research, all six soft skills—and especially perseverance, curiosity, and adaptability—directly relate to academic achievement (Credé et al., 2017; Durlak et al., 2011; Holliman et al., 2019; Lounsbury et al., 2009; Martin et al., 2012; von Stumm et al., 2011; Wagner & Ruch, 2015). Recent evidence suggests, however, that soft skills might not relate directly to academic achievement. Their effect would be mediated by other factors, such as SRL, scholastic motivation, and emotions (e.g., Chan et al., 2012; Muenks et al., 2017; Wolters & Hussain, 2015). Other studies highlighted the direct relation between students’ single soft skills and emotional, behavioral, and cognitive components of learning, which could fully mediate the effect of soft skills on academic achievement. This would be in line with the definition of soft skills as regulators of emotional, behavioral, and cognitive components of the person (Robles, 2012). These relations included achievement emotions (Dametto & Noronha, 2019; Robitschek & Keyes, 2009; Weber et al., 2016); motivation, in terms of self-efficacy, learning goals, and theories of intelligence (Martin et al., 2012; Muenks et al., 2018; Ruch et al., 2014); and SRL (Burns et al., 2018; Richards et al., 2013; Weisskirch, 2018). Taking this view, it was found that the six soft skills included in the WEF model compose a single factor, which is indirectly related to academic achievement through the mediation of SRL and motivation (Feraco et al., 2021). The authors did not include emotional aspects or students’ life satisfaction in their model, however, and studies considering soft skills together with other predictors of academic achievement are scarce, requesting further research in education.

Soft skills should promote an individual’s sense of fulfillment and wellbeing (Bruna et al., 2019; Peterson & Seligman, 2004). This relation has been found repeatedly across all age groups, and all the six soft skills considered have emerged as important correlates of life satisfaction (Bruna et al., 2019; Credé et al., 2017; Kashdan & Steger, 2007; Martin et al., 2012; Robitschek & Keyes, 2009; Stevic & Ward, 2008). The additional relation identified between soft skills and achievement emotions (e.g., Dametto & Noronha, 2019; Robitschek & Keyes, 2009; Weber et al., 2016) should further contribute to a stronger sense of life satisfaction (Hagenauer et al., 2018; Heffner & Antaramian, 2016; Karatzias et al., 2002). This makes soft skills worth examining further, taking a holistic approach to students, even though these skills might relate only indirectly to academic achievement.

The main aim of the present study was thus to examine how soft skills relate to academic achievement and life satisfaction, also taking other important learning-related factors into account (Ben-Eliyahu, 2019).

### Extracurricular activities

Given the international demand for soft skills development (European Commission, 2016; Pellegrino & Hilton, 2012), a complementary aim of this study was to explore whether students who participate in ECA have better soft skills (Feraco et al., 2021). Taking part in structured ECA is an important aspect of most school students’ lives (Feldman & Matjasko, 2005). It gives them a chance to experience challenging situations, widen their social networks, express and explore their identity, learn new skills, and develop personal qualities, such as soft skills (Eccles, 1999; Feldman & Matjasko, 2005; Khasanzyanova, 2017). There have been reports of ECA also having positive effects on factors such as academic achievement (Gilman & Huebner, 2006), SRL (Guilmette et al., 2019), self-efficacy (Marsh, 1992), positive and negative emotions at school, and life satisfaction (Gilman & Huebner, 2006; Guilmette et al., 2019; or see Shulruf, 2010 for a meta-analysis). Given these findings, the fact that students can usually choose freely whether to engage in ECA, and that schools might include ECA in their curricula, we believe it is worth considering their influence in a model of students’ academic achievement and wellbeing.

### Rationale of the study

This study aims to integrate different proven predictors of academic achievement and examine how they relate to each other and to both life satisfaction and academic achievement. Within a more classical model (Mega et al., 2014), we propose to include soft skills and ECA as potentially important aspects of a student’s life. Considered separately, all these factors have been previously found to correlate with both academic achievement and life satisfaction but examining how they relate to one another could result in a clearer picture of their direct and indirect effects on academic achievement and life satisfaction as part of a whole intraindividual system (Ben-Eliyahu, 2019).

Many authors have reported positive links between single soft skills and emotional, behavioral, and cognitive components of learning such as those we consider (e.g., Dametto & Noronha, 2019; Muenks et al., 2018; Richards et al., 2013; Weber et al., 2016; Weisskirch, 2018). If soft skills regulate these aspects, this should help students to reach their goals, and soft skills should have a mediated rather than any direct effect on academic achievement contrarily to what most of the previous literature suggests. Muenks et al. (2017), for example, support this hypothesis, having found that perseverance has much weaker direct effects on academic achievement once students’ motivation (or SRL, Wolters & Hussain, 2015) has been taken into account. The same seems to apply to curiosity after SRL has been accounted for (Chan et al., 2012), adaptability (Martin et al., 2012), or to soft skills in general (Feraco et al., 2021), but more work is needed to strengthen these results.

Soft skills should also be important to students’ life satisfaction (Bruna et al., 2019). Their influence should stem from a direct association, given that soft skills are general personal qualities that sustain individuals in any situation, not just at school, and an indirect association, mediated by achievement emotions, which are strongly related to life satisfaction (Hagenauer et al., 2018; Karatzias et al., 2002). This latter relation needs to be confirmed, however, bearing in mind that other aspects—such as SRL, motivation, and achievement emotions—could affect life satisfaction too. Since we hypothesize that the different soft skills have a similar pattern of relations with the other variables, we judge it plausible and informative to consider them as a single factor (as done previously, Feraco et al., 2021) to draw general theoretical conclusions on their role in the pattern of relations tested. Further studies and larger samples could precisely estimate the role of each of them.

Lastly, ECA could matter because they can promote school students’ soft skills and cognitive abilities (Eccles, 1999; Voyer & Jansen, 2017), support their life satisfaction (Gilman & Huebner, 2006), and influence factors such as academic achievement, SRL, motivation, and achievement emotions. That said we do not expect to find strong links between ECA and SRL, motivation, and achievement emotions after accounting for cognitive abilities and soft skills. This is because ECA do not directly regard school subjects or learning strategies, but they may improve students’ more general personal endowments (such as their soft skills and cognitive abilities), with a consequent impact on variables regarding their school life.

### Hypotheses

**Fig. 1:**
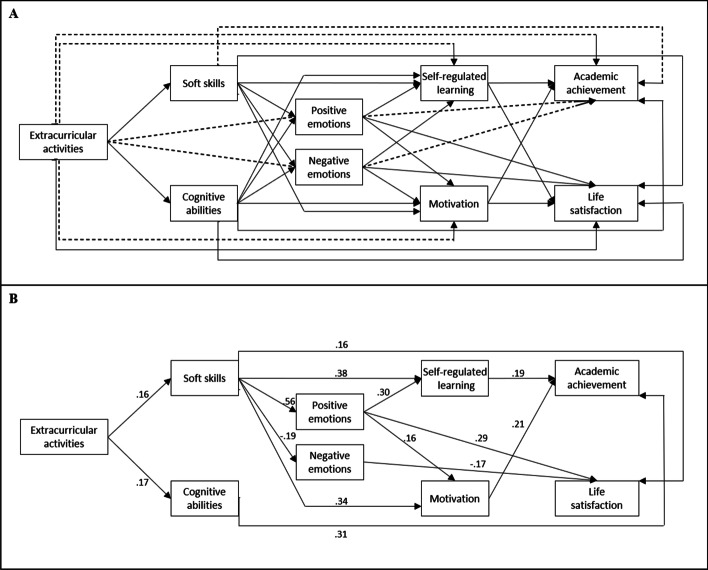
Representation of the fitted path analysis models (**A**), and standardized results for m2 (**B**). The dotted lines represent the paths whose priors differed in the two models. In “**B**,” only the paths associated with *β* > .09 in m2 are represented. Correlations are not shown

We advance hypotheses for each step of the path analysis outlined below (Figure 1) based on a Bayesian approach which request, to set plausible priors, carefully revising the literature to individuate and estimate the relations occurring between all the variables considered. Indeed, all hypothesized relations are supported by evidence in the literature and by a meta-analytical calculation of plausible effect sizes (i.e., priors). In eight cases, however—such as the direct relation between soft skills and academic achievement—we hypothesize a different relation from the one suggested by the meta-analytical priors and most of the previous literature. To be precise, we hypothesize that these eight relations are null when other variables are considered in the model at the same time. In other words, we assume that these relations are fully mediated by other variables. For this reason, we will compare two different models (one assuming the presence of the eight direct effects [m1], the other assuming that these effects are null [m2]) to test whether our hypotheses are confirmed.

The relations hypothesized are as follows:

*Academic achievement and life satisfaction—*we expect to find a small correlation between the two (Suldo et al., 2006).

*SRL and motivation—*we expect SRL and motivation to be positively related to academic achievement (Richardson et al., 2012) and to life satisfaction (especially motivation; Antaramian, 2017; Peters et al., 2018). Both SRL and motivation should also correlate (see Mega et al., 2014).

*Achievement emotions—*we expect positive and negative emotions to show respectively a positive and a negative direct association with SRL, motivation, and life satisfaction (Diener, 2012; Huang, 2011; Pekrun et al., 2007). We also expect them to be indirectly related (i.e., mediated by SRL and motivation) to academic achievement (Mega et al., 2014) and life satisfaction.

*Soft skills—*we expect soft skills to directly relate to achievement emotions, motivation, SRL, and life satisfaction (Feraco et al., 2021; Bruna et al., 2019; Weber et al., 2016). The relation between soft skills and academic achievement should be fully mediated by SRL, motivation, and achievement emotions (Chan et al., 2012; Muenks et al., 2017). Soft skills should also be indirectly associated with life satisfaction, SRL, and motivation through the mediation of achievement emotions. These relations are assumed to apply to all the soft skills, but—to avoid type 2 errors—we consider them primarily as a single factor, and then test the stability of the relations for all of them.

*Cognitive abilities—*we can expect cognitive abilities to be the strongest direct predictor of academic achievement (Roth et al., 2015). They should be mostly unrelated to the other factors, apart from a small positive association with motivation (Kriegbaum et al., 2018; Lavrijsen et al., 2021).

*Extracurricular activities*—the literature suggests that ECA could be related to every other variable considered, but authors only found them significantly related to soft skills, not to SRL or motivation, when they were all included in the same model. Soft skills should, in turn, indirectly support SRL and motivation. We expect the same to apply to achievement emotions, with ECA indirectly relating to achievement emotions through the mediation of soft skills. Finally, we expect ECA to be positively linked to life satisfaction (Gilman, 2001; Gilman & Huebner, 2006), and cognitive abilities, as seen in studies on practicing sports and music (Schellenberg, 2004; Voyer & Jansen, 2017).

## Method

### Participants

The study involved 603 students ranging from 11 to 18 years old (in Italian school grades 6 to 12; M_age_ = 13.53, SD = 1.91), who participated on a voluntary basis after their consent form was signed. Another 56 students participated in the study but did not complete all the tasks and were consequently excluded a priori.

### Measures

All measures showed a good internal consistency (omega coefficients are reported in Table S1 in Supplementary materials).

#### Extracurricular activities

An ad hoc questionnaire was used to assess the amount of structured and regular activities that students attended in their out-of-school time. Three types of activity were selected according to previous studies (Feraco et al., 2021; Shulruf, 2010): sport, music, and associative activities (e.g., scouting, volunteering). Participants were asked to indicate how many years they had been engaging in each type of activity. The sum of the years for all three was considered the amount of a student’s ECA.

#### Soft skills

The soft skills questionnaire (Feraco et al., 2021) measures students’ levels of the six soft skills belonging to the personal qualities branch of the World Economic Forum model (, 2016): (i) *adaptability* (e.g., “I’m scared by situations that are new to me.”); (ii) *curiosity* (e.g., “Whenever I see something new, I try to understand what it is.”); (iii) *initiative* (e.g., “If a decision has to be taken, I take it.”); (iv) *perseverance* (e.g., “Faced with a difficult situations, I don’t give up.”); (v) *social awareness* (e.g., “It’s important that all people be treated equally.”); (vi) *leadership* (e.g., “I can take the lead in team work.”). Each subscale includes 6 items, except for the leadership scale, which has 4. Answers are given on a 6-point Likert scale. The questionnaire has shown a good factorial validity, as measured by confirmatory factor analysis, and the total soft skills score has been found reliable (Feraco et al., 2021). The validity of the soft skill second-order factor was also confirmed on our sample (see Measurement models section in Supplemental Materials). We computed the average of each subscale and the mean of the averages for the total score.

#### Achievement emotions

The *Positive and Negative Affect Schedule* (PANAS, Watson et al., 1988; Italian adaptation by Terraciano et al., 2003) was used to measure emotions at school. It measures positive and negative emotions (10 items each) on a 5-point Likert scale indicating how much students experienced each of the 20 emotions at school during the previous weeks. Average scores for positive (PA) and negative (NA) emotions are calculated separately.

#### Motivation to learn

The *Scholastic Motivation questionnaire* (SM, De Beni et al., 2014) measures academic motivations in terms of (i) *incremental theory of intelligence* (e.g., “You can learn new things, but you can’t change your intelligence.”); (ii) *mastery learning goals* (e.g., “It’s more important to me to learn things than to get good grades.”); and (iii) *self-efficacy* (e.g., “How do you rate your study skills?”). The questionnaire includes 13 items (5 for self-efficacy, and 4 for theory of intelligence and learning goals) on a 5-point Likert scale. As done previously in Mega et al. (2014), we considered the three subscales as a single indicator of motivation. The validity of the academic motivation second-order factor was confirmed on our sample (see Measurement models section in Supplemental Materials). We computed the average of the means for each subscale after reversing the negative items.

#### Self-regulated learning

The *Self-Regulated Learning Questionnaire* (SRL, De Beni et al., 2014) measures SRL in terms of *elaboration* (e.g., “When I study, I try to literally repeat the textbook.”); *strategies* (e.g., “When I study for an exam, I wonder what my teacher considers important.”); *metacognition* (e.g., “If a test goes badly, I try to understand why it happened.”); *organization* (e.g., “I usually know how to organize my studies so that I still have time for my hobbies.”); and *self-evaluation* (e.g., “I understand when the task I have to do is easy or difficult for me.”). It is composed of 50 items (10 for each aspect) on a 5-point Likert scale. After reversing the negative items, we calculated the average score.

#### Cognitive abilities

The *Culture-Free Intelligence Test* (Cattell, 1940) measures fluid intelligence. It consists of 46 items divided in 4 different time-limited subtasks (series completion, odd-one-out, matrices, and topology) that present items in order of difficulty. One point is awarded for each correct answer and the total score is calculated as the sum of all the subtask scores.

#### Academic achievement

##### Grades

The Italian school system provides for summary grades twice a year (February and June) on a 10-point scale, where 6 is a pass. We collected students’ grades in February for the four most representative subjects that are common to all academic years and types of school: Italian, foreign language (English), math, and science. Each student’s average grade was calculated as a measure of their academic achievement.

#### Life satisfaction

The *Satisfaction With Life Scale* (SWLS, Diener et al., 1985; Italian adaptation by Di Fabio & Gori, 2016) measures overall life satisfaction with 5 items (e.g., “The condition of my life are excellent.”) on a 7-point Likert scale. The average score was calculated.

### Procedure

We contacted schools and scheduled a meeting to discuss and explain the project with those interested in taking part. Consent forms were distributed by the schools to parents who signed them before data collection (January to February 2020).

Two collective sessions lasting 1 h were organized during school hours. A trained psychologist administered the questionnaires and tasks in the presence of the class teachers. Participants always started by providing personal information and ECA. The tasks were counterbalanced across classes. For the cognitive tasks, the experimenter read the instructions and completed the practice items together with the students before letting them answer. Students were stopped when the time limits were reached. For the questionnaires, the experimenter asked students to read the instructions carefully and told them that there were no right or wrong answers. At the end of February, the schools provided us with the grades obtained by each participant in math, science, foreign language (English), and Italian.

### Data analysis

All analyses were run using R (R Core Team, 2019) after standardizing the scores by participants’ school year. Table S1 shows the means, standard deviations, omega coefficients, and correlations between all variables.

#### Prior specification

We adopted a Bayesian approach for the path analysis in order to (i) include previous evidence from the literature (prior beliefs) in the model, leading to more precise estimates of the effects, and (ii) formulate, estimate, and compare precise alternative hypotheses in terms of probability distributions around a plausible effect (McElreath, 2016). Before running the path analysis, we thus estimated a prior distribution for the relationship existing between each pair of variables considered. Then two models were fitted under different prior assumptions (hypotheses) and compared using the R package “blavaan” (Merkle & Rosseel, 2018). The comparison enabled us to see which hypothesis fitted the data better.

In Bayesian analyses, “prior” and “posterior” (probability) distributions are fundamentals. Priors represent what we already know about the magnitude of given parameters (e.g., correlation coefficient) and its related uncertainty, before collecting data. Posteriors represent how prior probability distributions change after new data are analyzed, so that we can compare what we thought before with the updated results (McElreath, 2016).

To set plausible priors, we searched for existing meta-analytical results, papers of which we were already aware, other related papers, and papers citing them. Available correlation results were transformed into Fisher’s *Z* values (see Borenstein et al., 2009 guidelines), and meta-analyzed using the R package “metafor” (Viechtbauer, 2010). For the variables belonging to second-order factors, we calculated the average of the resulting meta-analytical *Z* values between each of the variables comprising the second-order factor and the related variable to estimate a plausible second-order prior. Finally, we transformed the *Z* values back into *r* coefficients and used the latter as priors for the standardized path coefficients. Three different kinds of prior were ultimately used:

- Weak (SD = .40) for the estimates calculated on a small number of effects
- Moderate (SD = .20) for all the other prior estimates in line with the hypotheses
- Strong (SD = .05) for all the prior estimates that contradicted our hypotheses

For example, a moderately informative prior *N*(.20, .20) is centered on .20 and has most of its density distribution (95%) ranging between −.17 and .60. A similar but strongly informative prior *N*(.20, .05) is still centered on .20, but has most of its density distribution (95%) ranging between .10 and .30, constraining the posterior distribution to a greater degree. See Table 1 for the priors used, or the Supplementary materials for a more detailed description of this process and Table S2.

**Table 1:** Posterior estimates of the direct relations for the two models fitted

Path	Model 1		Model 2		
	β	HPDI 95%	β	HPDI 95%	Prior
SRL→AA	.14	[.05 .22]	.19	[.10, .28]	*N*(.17,.20)
SM→AA	.19	[.10, .26]	.21	[.14, .29]	*N*(.19,.20)
PA→AA	.03	[−.03, .09]	−.00	[−.06, .06]	*N*(.12,.05)^a^
NA→AA	−.10	[−.16, −.04]	−.05	[−.11, .01]	*N*(−.16,.05)^a^
CA→AA	.29	[.22, .35]	.31	[.24, .37]	*N*(.54,.20)
SS→AA	.02	[−.04, .09]	−.05	[−.11, .02]	*N*(.19,.05)^a^
ECA→AA	.11	[.06, .17]	.07	[.02, .13]	*N*(.13,.05)^a^
SRL→LS	.00	[−.09, .09]	.00	[−.08, .10]	*N*(.17,.40)
SM→LS	.05	[−.03, .13]	.05	[−.04, .13]	*N*(.33,.20)
PA→LS	.29	[.21, .39]	.29	[.20, .37]	*N*(.40,.20)
NA→LS	−.18	[−.24, −.10]	−.17	[−.24, −.10]	*N*(−.35,.20)
CA→LS	−.02	[−.09, .05]	−.02	[−.09, .05]	*N*(.10,.20)
SS→LS	.16	[.07, .25]	.16	[.06, .25]	*N*(.36,.20)
ECA→LS	.06	[−.01, .13]	.06	[−.01, .13]	*N*(.17,.20)
PA→SRL	.30	[.23, .37]	.30	[.22, .37]	*N*(.36,.20)
NA→SRL	−.04	[−.10, .03]	−.04	[−.10, .03]	*N*(−.27,.20)
CA→SRL	.01	[−.05, .08]	.02	[−.05, .08]	*N*(.10,.40)
SS→SRL	.37	[.29, .44]	.38	[.30, .45]	*N*(.39,.20)
ECA→SRL	.09	[.04, .14]	.04	[−.01, .09]	*N*(.15,.05)^a^
PA→SM	.16	[.08, .24]	.16	[.08, .24]	*N*(.32,.20)
NA→SM	−.10	[−.16, −.02]	−.09	[−.17, −.03]	*N*(−.12,.20)
CA→SM	.04	[−.03, .11]	.05	[−.02, .12]	*N*(.17,.20)
SS→SM	.34	[.25, .42]	.34	[.26, .42]	*N*(.26,.20)
ECA→SM	.03	[−.02, .09]	−.00	[−.06, .05]	*N*(.07,.05)^a^
CA→PA	−.09	[−.15, −.02]	−.07	[−.14, −.00]	*N*(.12,.40)
SS→PA	.54	[.48, .59]	.56	[.51, .62]	*N*(.38,.20)
ECA→PA	.14	[.08, .19]	.00	[−.06, .06]	*N*(.40,.05)^a^
CA→NA	−.07	[−.15, .01]	−.08	[−.16, −.00]	*N*(.09,.40)
SS→NA	−.18	[−.26, −.10]	−.19	[−.26, −.11]	*N*(−.19,.20)
ECA→NA	−.03	[−.09, .03]	.02	[−.04, .09]	*N*(−.12,.05)^a^
ECA→CA	.17	[.09, .24]	.17	[.09, .24]	*N*(.02,.40)
ECA→SS	.16	[.09, .24]	.16	[.09, .24]	*N*(.12,.20)

Note. ^a^The prior in m2 was *N*(.00, .05)

*HPDI*, higher posterior density interval; *AA*, academic achievement; *LS*, life satisfaction; *SRL*, self-regulated learning; *SM*, scholastic motivation; *PA*, positive emotions; *NA*, negative emotions; *CA*, cognitive abilities; *SS*, soft skills; *ECA*, extracurricular activities

#### Path analysis

**Fig. 2:**
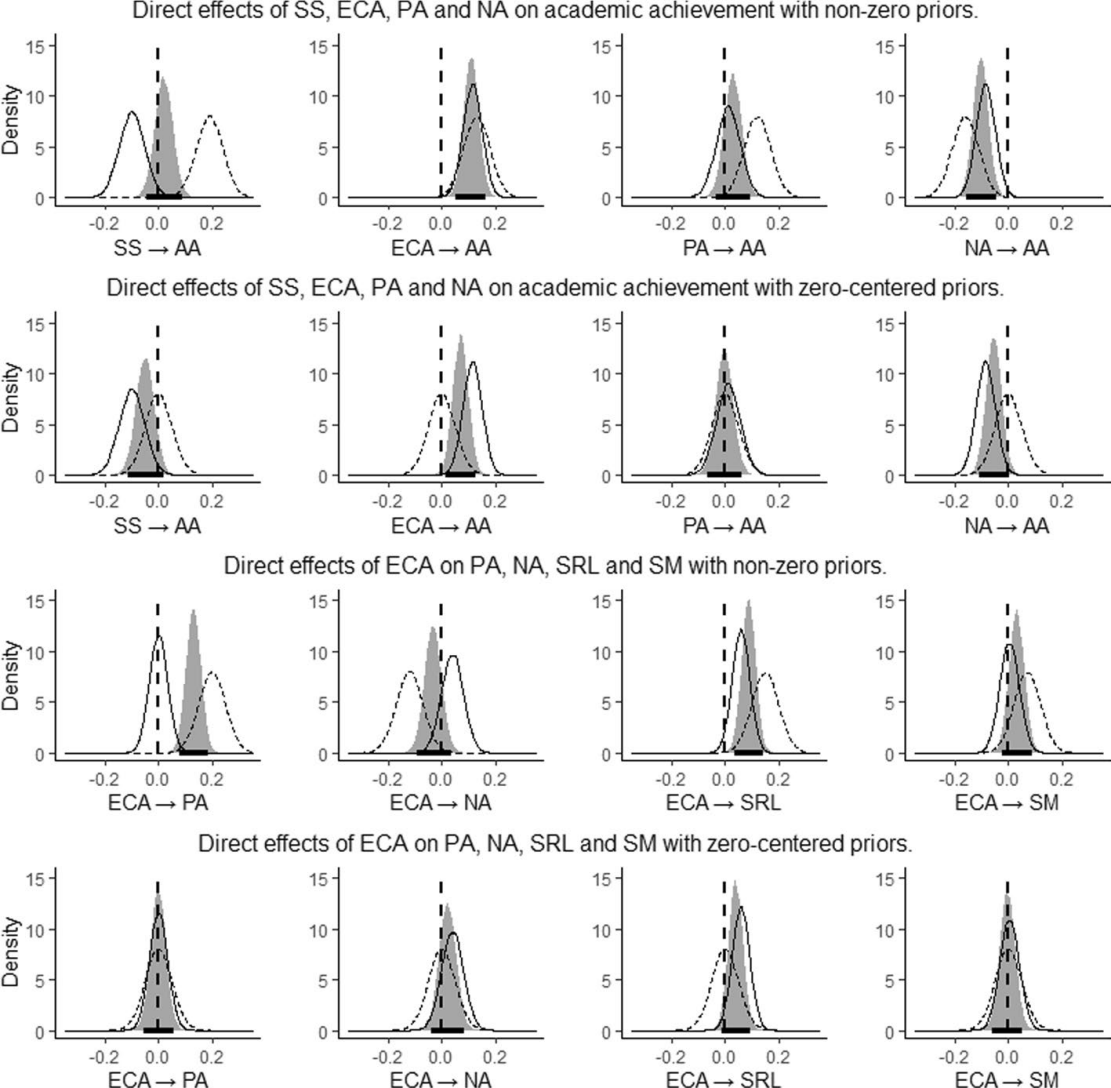
Prior (dotted curve), likelihood (continuous line curve), and posterior (gray curve) distributions of the eight effects specified with different priors. Rows 1 and 3 (m1) show the effects obtained with non-zero priors. Rows 2 and 4 (m2) show the effects obtained with zero-centered priors. The thick horizontal line represents higher posterior density intervals. Note. AA = academic achievement; LS = life satisfaction; SRL = self-regulated learning; SM = scholastic motivation; PA = positive emotions; NA = negative emotions; CA = cognitive abilities; SS = soft skills; ECA = extracurricular activities

We estimated and compared two full path analysis models, which differed only in terms of the priors specified for eight effects (see Figure 1 and Figure 2 for a visual representation of the model). Four chains of 10,000 iterations were run for each model, ending with a total of 40,000 used iterations per model. The two models differed only in the priors specified on the following eight relations (see Figure 2 and Table 1): direct relations of soft skills, PA, NA, and ECA with academic achievement; and direct relations of ECA with motivation, SRL, PA, and NA. In the first model (m1), for each of these eight relations, we set a strongly informative prior (SD = .05) centered on the estimate calculated in the previous section (e.g., *N*[.19, .05] on the association between soft skill and academic achievement). In the second model (m2), the priors for the same relations were centered on 0, thereby representing a different hypothesis (e.g., *N*[.00, .05] on the relation between soft skill and academic achievement). All other priors were identical (see Table 1). This procedure enabled us to compare the hypothesis of a null effect (m2) with the hypothesis of the presence of an effect, as suggested by previous literature (m1). The two models were compared using the widely applicable information criterion (WAIC; Watanabe, 2010) and the Laplace approximation to log-Bayes factors (Raftery, 1993). The results show that m2 was preferable (ΔWAIC = 24.74; logBF = 33.92), so the effect for the eight relations is plausibly zero, or practically equivalent to zero. See Figure 2 for a graphical representation of the overlap between the different priors of the two models and the data considered. Bayesian fit indices were calculated for m2 confirming that m2 explains the data well (RMSEA = .04, CFI =.99, TLI = 1.04).

The final model was run again, adding one soft skill at a time instead of the aggregate factor to ensure that the pattern of relations remained stable.

## Results

Given that m2 proved preferable to m1, we present its posterior predictions. We considered the effects as practically equivalent to 0 when the higher posterior density intervals (HPDI) comprised 0, or the direct effect was lower than .10 (Kruschke & Liddell, 2018). The posterior predictions for m2 largely confirmed our hypotheses (see Table 1, Figure 1, and Figure S1 in the Supplementary materials for the detailed results obtained with the two models). SRL, motivation, and cognitive abilities showed a positive direct relation with academic achievement, but PA, NA, soft skills, or ECA did not. PA and soft skills showed a positive direct relation with life satisfaction, and NA showed a negative direct relation with life satisfaction, while SRL, motivation, cognitive abilities, and ECA showed only a negligible association with it. PA and soft skills were positively related to SRL and motivation, while NA showed a small relation with motivation, but not with SRL. Cognitive abilities and ECA showed no noteworthy association with either SRL or motivation. Soft skills were the only variable relating positively to PA and negatively to NA. Finally, ECA were related to both cognitive abilities and soft skills. The estimated correlations between academic achievement and life satisfaction and between PA and NA were very small. On the other hand, there were associations between SRL and motivation and between soft skills and cognitive abilities, though the latter correlation was so small as to be negligible.

As expected, the analysis of the indirect effects showed that soft skills work as a mediator between ECA and PA, NA, motivation, and SRL. The effect of soft skills on academic achievement was also mediated by SRL and motivation, and the effect of soft skills on life satisfaction was not only direct, but also mediated by PA and NA. Finally, PA (but not NA) was indirectly related to academic achievement through the mediation of SRL and motivation (see Table S3 in the Supplementary materials for the list of all the indirect effects).

This pattern of relations was generally confirmed for the analysis run with single soft skills instead of the aggregate soft skill factors (see Figure S2 in the Supplemental materials). In particular, none of them showed a positive direct relation with academic achievement (*β* = [−.08; .02]), but they did show positive relations with life satisfaction (except for social awareness; *β* = [.05; .19]), PA (*β* = [.28; .51]), scholastic motivation (*β* = [.08; .41]), and SRL (*β* = [.07; .40]) and a negative relation with NA (except for social awareness; *β* = [−.13; −.31]). ECA was also positively related with each of the six soft skills (*β* = [.05; .17]).

## Discussion

Soft skills are important personal characteristics that promote an individual’s wellbeing and success, but their role in school is not consistently examined. With this study, we aimed to combine relevant soft skills within an exhaustive intraindividual model of learning that included ECA, achievement emotions, cognitive abilities, SRL, and motivation in relation to academic achievement and life satisfaction. Such a broad model adds to our understanding of the pattern of relations existing between these variables that, taken one at a time, have often been found important to both academic achievement and life satisfaction, but that also interact together. Our main assumption was that soft skills directly regulate students’ behavioral, cognitive, and emotional aspects of learning, but not their academic achievement, which should be fully mediated by SRL, motivation, and achievement emotions. On the other hand, soft skills should play an important part in supporting students’ life satisfaction, both directly and indirectly, making them important factors contributing to students’ wellbeing and success.

Besides the effect of achievement emotions, SRL, motivation, and cognitive abilities on academic achievement—that was clearly in line with previous evidence and with the control-value theory (Feraco et al., 2021; Kriegbaum et al., 2018; Lavrijsen et al., 2021; Mega et al., 2014; Pekrun et al., 2007)—the importance of soft skills to academic achievement emerged clearly when we considered their association with the above-mentioned factors. As expected, soft skills showed positive relations with all these factors: students with stronger soft skills are likely to be more motivated to study, to use more and better SRL processes, and to experience positive feelings at school. In other words, soft skills in the scholastic context regulate school students’ behavior, thoughts, and emotions (Martin et al., 2012; Park et al., 2004) consequently favoring academic achievement (Feraco et al., 2021). Previous studies (e.g., Muenks et al., 2017; Richards et al., 2013) had already found little or no relation between specific soft skills and academic achievement after accounting for other school-related variables. Based on this evidence, soft skills should work similarly: being curious, perseverant, adaptable, and aware of others or being able to lead a group or take the initiative does lead to greater success in specific areas, such as academic achievement, but only when these characteristics promote functional behaviors, emotions, and thoughts. Otherwise, for instance, someone might take the initiative without carefully considering the situation or which strategies to adopt (SRL at school), thereby lowering their chances of success.

As mentioned at the start of this paper, there is more to a student’s life than academic achievement, and we believe it is important to understand which variables may play a role in both students’ success and satisfaction (Suldo et al., 2006). Our results regarding life satisfaction partially contradict previous findings on how SRL and motivation relate to life satisfaction, but they also confirm that the two dependent variables are largely unrelated. It would therefore be of primary importance to understand how to promote both (Weber et al., 2016). SRL and academic motivation had previously been found positively related to students’ life satisfaction (Antaramian, 2017), but our study did not confirm this link suggesting that their role might be limited to learning outcomes, or that SRL and academic motivation are narrow factors that directly entail only academic variables, such as achievement or eventually school satisfaction, but that do not extend their influence on not-scholastic ones. No relations were found also between life satisfaction and cognitive abilities (Suldo et al., 2006). This is in line with a recent meta-analysis showing negligible relations between cognitive abilities and both life and job satisfaction (Gonzalez-Mulé et al., 2017) and suggests that people do a cognitive evaluation of their own life factoring out their “innate” abilities, or, in other words, that they evaluate what they have done respect to what their abilities could permit them. Differently, we did confirm a positive relation between achievement emotions and life satisfaction. Like general emotions, achievement emotions are a major component of life satisfaction (Diener et al., 1985; Hagenauer et al., 2018; Heffner & Antaramian, 2016; Karatzias et al., 2002) and, in our case, they were the only scholastic-related variable directly influencing students’ wellbeing. Here again, the role of soft skills seems to be important: they were the only other variable considered that directly related to life satisfaction. This is presumably because soft skills refer to individuals’ personal qualities that describe the person in any situation, not only at school. These skills induce them to adopt a more functional behavior and generally to feel more positive and fewer negative emotions (Park et al., 2004) making the association between soft skills and life satisfaction both direct and mediated by students’ achievement emotions. This would further support their contribution to a more flourishing life in general.

The positive association between soft skills with ECA suggests that ECA might modulate soft skills (Feraco et al., 2021; Eccles, 1999; Khasanzyanova, 2017). Indeed, ECA give students the opportunity to face challenges, interact with others, and explore their own identity (Eccles et al., 2003). As expected, ECA was consistently associated with soft skills and cognitive abilities (Voyer & Jansen, 2017), but not with the scholastic-specific variables considered. These relations were only mediated indirectly by soft skills (Feraco et al., 2021). Finally, the link between ECA and life satisfaction was close to zero, and the effect of these activities was again mediated only by soft skills. This unexpected result may be because we considered how many years a student had engaged in ECA, whereas asking simply whether or not they had ever done so might have revealed a direct association with students’ life satisfaction (Gilman, 2001; Gilman & Huebner, 2006). In fact, it might be that there is no linear relationship between the years of practice of an activity with one’s life satisfaction, but the latter might be more easily influenced by the actual practice of something that the person value as important and that fulfill his or her life with its presence and not with the ability that a student has in practicing a sport, for example. This is also in line with what we found in relation with academic achievement or cognitive abilities.

In general, our results support the importance of soft skills in favoring students’ life satisfaction (both directly and indirectly), achievement emotions, and academic achievement (through the mediation of SRL and motivation). On the other hand, classical predictors of academic achievement (i.e., SRL, motivation, and cognitive abilities) only influenced students’ academic achievement, but not life satisfaction. These results are in line with the recommendations of several international organizations and institutions (European Commission, 2016; MIUR, 2018; Pellegrino & Hilton, 2012) that underscore the importance of focusing more on students’ soft skills (among other things) to promote school students’ wellbeing and more functional approach to learning. Nurturing their soft skills will also improve their opportunities when they enter the job market (Heckman & Kautz, 2012) and their life satisfaction in adulthood (Bruna et al., 2019). Such an approach is even more justified by the fact that soft skills are malleable. Just engaging in ECA seems to relate to their development in adolescence. While targeted interventions for soft skills could be implemented during school hours, by training teachers and educators to adapt their teaching methods, or by working directly with the students, schools may also consider ECA as a plausible way to shape students’ soft skills.

Our study has some limitations, however. First of all, the cross-sectional nature of the study prevents us from inferring any causal relations among the variables. Soft skills, even if the measure resulted statistically reliable in our sample, were measured with questionnaires that were not validated following a complete scientific approach possible mining the reliability of the results. We also treated soft skills and motivation as single factors: this gave us the opportunity to describe and test how soft skills should generally relate to the different factors considered, but means we cannot fully extend our results to the single skills, or compare their relative importance (though exploratory findings suggest that their individual roles are similar).

## Conclusions

Our findings support the importance of soft skills in school students. These skills were found positively related not only to SRL, motivation, and achievement emotions but also to life satisfaction. Soft skills thus support students’ wellbeing, as well as their academic achievement, whereas SRL and motivation show no noteworthy association with life satisfaction (their importance is limited to school). Finally, ECA emerged as a plausible practical way to develop students’ soft skills and cognitive abilities—although our findings were cross-sectional, and therefore cannot indicate any causal relation. Our results indicate that it is important to consider an integrated intraindividual model of learning (Ben-Eliyahu, 2019) in order to shed more light on the real role of each factor influencing school students’ academic achievement and satisfaction.

## Supplementary Information

Below is the link to the electronic supplementary material.

Supplementary file1 (DOCX 3725 kb)

## Data Availability

Data are available on Figshare. DOI: 10.6084/m9.figshare.13182401.
