# Supplementary material for: An integrated model of school students’ academic achievement and life satisfaction. Linking soft skills, extracurricular activities, self-regulated learning, motivation, and emotions
Source: Eur J Psychol Educ. 2022 Jan 28;38(1):109–30. doi: 10.1007/s10212-022-00601-4 (PMC8795749; doi:10.1007/s10212-022-00601-4)
Supplement: Supplementary file 1 — Supplementary file1 (DOCX 3725 kb) [file 10212_2022_601_MOESM1_ESM.docx]

**Supplementary materials**

An integrated model of school students’ academic achievement and life satisfaction. Linking soft skills, extracurricular activities, self-regulated learning, motivation, and emotions

These materials include:

- The analysis of the factorial structure of the two second-order factors of soft skills and academic motivation (Measurement models for soft skills and motivation);
- A more detailed description of the meta-analytical work done for the purpose of estimating the priors (Supplementary description of the prior specification process);
- Supplementary tables (S1 includes means, standard deviations, omega, and correlations between all study variables, S2 lists all the studies, and the effects considered to estimate each prior; S3 shows all the indirect effects of the path analysis models);
- Supplementary figures (posterior, likelihood, prior distributions for each estimate of model 2, and results for each soft skill);
- References to all the papers consulted in the prior estimation process.

## Measurement models for soft skills and motivation

Before running the path analysis, we ascertained that the six soft skill subscales and the three motivation subscales converge into two second-order factors, as previously reported by Feraco et al. (2021) and Mega et al. (2014), respectively. A confirmatory factor analysis (CFA) was run to validate the use of the aggregate factors in the subsequent path analysis (Schreiber, 2008). This enabled us to reduce the number of parameters to estimate in the path analysis and avoid running an over-parametrized model (although the consistency of the effect for the six soft skills is explored).

The CFA for the soft skills factor included the 34 items as observed variables, and 6 latent factors (i.e., adaptability, curiosity, initiative, leadership, perseverance, and social awareness). The CFA for the scholastic motivation factor included the 14 items as observed variables, and three latent factors (i.e., theories of intelligence, self-efficacy, and learning goals). As suggested by Schermelleh-Engel et al. (2003), the goodness of the models’ fit to the data was assessed using multiple indexes: chi-square, the comparative fit index (CFI), the non-normed fit index (NNFI), and the root-mean-square error of approximation (RMSEA). The models showed acceptable fit indexes (*χ^2^*(521, N = 600) = 2610.39, *p* < .001, CFI = .93, NNFI = .93, RMSEA = .08, 90% confidence interval for RMSEA [.08, .09], for soft skills; *χ^2^*(62, N = 600) = 404.76, *p* < .001, CFI = .95, NNFI = .93, RMSEA = .10, 90% confidence interval for RMSEA [.09, .11], for scholastic motivation) and significant factor loadings (*p* < .001), with the mean factor loading of the second-order factors amounting to .79 and .48, respectively. A multi-group confirmatory factor analysis was also run using diagonally weighted least squares (DWLS) to ensure that the measurement model was the same for upper secondary school (N = 266) and lower secondary school students (N = 334). The results showed a strong scalar invariance for both the models (equality of factor loadings and intercepts): *χ^2^*(461, N = 600) = 3877.89, *p* < .001, CFI = .92, NNFI = .93, RMSEA = .09, 90% confidence interval for RMSEA [.08, .09], for soft skills, *χ^2^*(171, N = 600) = 594.504, *p* < .001, CFI = .94, NNFI = .94, RMSEA = .09, 90% confidence interval for RMSEA [.08, .19], for scholastic motivation.

**Supplementary description of the prior specification process**

Here, we describe the prior specification process for each of the 36 relations inspected in more detail. A light meta-analytical approach was adopted: instead of performing a full literature review for each relation, we only searched for papers of which we were already aware, other related papers, and papers citing them, or already done meta-analysis. For each relation, we indicate the number of effects and studies retrieved, and the magnitude of each one, together with the associated confidence intervals (CI). For the purpose of our analysis, however, we only used the magnitude of the effect as a prior, adding preset standard errors based on how accurate the estimation was, and whether we hypothesized a different effect for it (see the Data analysis section). The priors ultimately used are presented in Table 1 in the paper.

*Soft skills and self-regulated learning*

The relation between soft skills and SRL has been studied in particular as concerns curiosity and perseverance. We retrieved 16 effect sizes from 7 different studies that showed a positive relation between self-regulated learning and adaptability (z = .43, CI [.07, .78]), curiosity (z = .40, CI [.22, .58]), initiative (z = .44, CI [.35, .54]), leadership (z = .31, CI [.22, .40]), perseverance (z = .50, CI [.43, .60]), and social awareness (z = .40, CI [.31, .49]). The mean of the six values obtained was computed (mean z = .42) and converted into *r* (*r* = .39).

*Soft skills and motivation*

The prior on the relation between soft skills and motivation includes the relations between each soft skill and the three aspects of motivation considered (i.e., self-efficacy, learning goals, and incremental theories of intelligence). We retrieved 64 effect sizes (25 for self-efficacy; 21 for learning goals; 18 for incremental theories of intelligence) from 21 published papers. Self-efficacy was found positively related with adaptability (z = .47, CI [.29, .67]), curiosity (z = .38, CI [.33, .44]), initiative (z = .24, CI [.16, .33]), leadership (z = .30, CI [.10, .49]), perseverance (from the meta-analysis by Credé et al., 2017, z = .46, CI for r [.28, .57]), and social awareness (z = .32, CI [-.16, .80]); and the mean of the six values obtained was computed (mean z = .36). Learning goals showed a positive relation with adaptability (z = .39, CI [.25, .53]), curiosity (z = .47, CI [.43, .52]), initiative (z = .22, CI [.13, .32]), leadership (z = .18, CI [-03., .40]), perseverance (z = .38, CI [.29, .47]), and social awareness (z = .28, CI [.20, .36]); and the mean of the six values obtained was computed (mean z = .32). Finally, incremental theories of intelligence showed relations with adaptability (z = .24, CI [.07, .41]), curiosity (z = .12, CI [.03, .21]), initiative (z = .17, CI [.08, .26]), leadership (z = .01, CI [-.08, .10]), perseverance (z = .23, CI [.13, .34]), and social awareness (z = -.01, CI [-.10, .08]); and the mean of the six values obtained was computed (mean z = .13). Then the three means were averaged (mean z = .27) and converted into *r* (*r* = .26).

*Soft skills and positive emotions*

We retrieved 27 correlation effects for the single soft skills and positive emotions from 15 published papers. The meta-analytical results showed that positive emotions were positively related to adaptability (z = .21, CI [.14, .29]), curiosity (z = .42, CI [.38, .47]), initiative (z = .40, CI [.31, .49]), leadership (z = .38, CI [.31, .45]), perseverance (z = .50, CI [.40, .60]), and social awareness (z = .46, CI [.30, .62]). The mean of the six values obtained was computed (mean z = .40) and converted into *r* (*r* = .38).

*Soft skills and negative emotions*

We retrieved 24 correlation effects for the single soft skills and negative emotions from 12 published papers, one of which was a meta-analysis (Weigold et al., 2020). The meta-analytical results showed a negative relation between negative emotions and adaptability (z = -.16, CI [-.26, -.06]), curiosity (z = -.14, CI [-.21, -.07]), initiative (from a meta-analysis by Weigold et al., 2020, z = -.25, CI for *r* [-.39, -.01]), leadership (z = -.16, CI [-.23, -.10]), perseverance (z = -.18, CI [-.25, -.11]), and social awareness (z = -.25, CI [-.31, -.19]). The mean of the six values obtained was computed (mean z = -.19) and converted into *r* (*r* = -.19).

*Soft skills and extracurricular activities*

Only two studies were retrieved, for a total of 7 effect sizes, for the purposes of specifying the prior of the relation between soft skills and ECA. We found the following correlations between ECA and adaptability (z = .08, CI [-.01, .17]), curiosity (z = .06, CI [-.03, .15]), initiative (z = .19, CI [.10, .29]), leadership (z = .19, CI [.10, .29]), perseverance (z = .15, CI [-.02, .33]), and social awareness (z = .08, CI [-.01, .17]). The mean of the six values obtained was computed (mean z = .13) and converted into *r* (*r* = .12).

*Soft skills and cognitive abilities*

Seventeen effect sizes were retrieved from 4 studies and two meta-analysis (Credé et al., 2017; von Stumm et al., 2011). We found the following correlations between cognitive abilities and adaptability (z = .09, CI [.04, .14]), curiosity (from a meta-analysis by von Stumm et al., 2011, z = .22), initiative (z = .19, CI [.01, .36]), leadership (z = -.06, CI [-.15, .03]), perseverance (from a meta-analysis by Credé et al., 2017, z = -.01, CI for *r* [-.06, .04]), and social awareness (z = .01, CI [-.08, .10]). The mean of the six values obtained was computed (mean z = .07) and converted into *r* (*r* = .07).

*Soft skills and life satisfaction*

We retrieved 119 effects (112 from a meta-analysis by Bruna et al., 2019) for the relation between the six soft skills and life satisfaction. The following correlations were found life satisfaction and adaptability (z = .44, CI [.25, .64]), curiosity (from a meta-analysis by Bruna et al., 2019, z = .42, CI for *r* [.38, .42]), initiative (z = .53, CI [.31, .75]), leadership (from the meta-analysis by Bruna et al., 2019, z = .24, CI for *r* [.20, .29]), perseverance (from the meta-analysis by Bruna et al., 2019, z = .33, CI for *r* [.26, .37]), and social awareness (from the meta-analysis by Bruna et al., 2019, z = .30, CI for *r* [.26, .32]). The mean of the six values obtained was computed (mean z = .38) and converted into *r* (*r* = .36).

*Soft skills and academic achievement*

One of the key aspects of the study was the direct relationship found between soft skills and academic achievement. We retrieved 33 effects (16 of them from meta analyses by Credé et al., 2017, and von Stumm et al., 2011, and 17 from 10 different studies). All of the soft skills showed a positive association with academic achievement. In particular, academic achievement related to adaptability (z = .18, CI [.10, .26]), curiosity (from a meta-analysis by von Stumm et al., 2011, z = .33), initiative (z = .09, CI [-.02, .18]), leadership (z = .13, CI [.06, .19]), perseverance (from a meta-analysis by Credé et al., 2017, z = .27, CI for *r* [.11, .41]), and social awareness (z = .13, CI [.08, .17]). The mean of the six values obtained was computed (mean z = .19) and converted into *r* (*r* = .19).

*Self-regulated learning and motivation*

We retrieved 46 effect sizes (35 of them from the meta-analysis by Burnette et al., 2012) for the relations between SRL and the three aspects of motivation considered (i.e., self-efficacy, learning goals, and incremental theories of intelligence). SRL was positively related to self-efficacy (z = .41, CI [.35, .47]), learning goals (z = .25, CI [.17, .33]) and incremental theories of intelligence (from the meta-analysis by Burnette et al., 2012; z = .13, CI for *r* [.16, .29]). The mean of the three values obtained was computed (mean z = .30) and converted into *r* (*r* = .29).

*Self-regulated learning and positive emotions*

We retrieved 27 effect sizes from 5 studies for the correlation between SRL and PA. The meta-analytical results showed a z value = .37 (CI [.33, .41]), which was converted into *r* (*r* = .36).

*Self-regulated learning and negative emotions*

We retrieved 24 effect sizes from 5 studies for the correlation between SRL and NA. The meta-analytical results showed a z value = -.28 (CI [-.31, -.26]), which was converted into *r* (*r* = -.27).

*Self-regulated learning and extracurricular activities*

We retrieved 6 effect sizes from two studies for the correlation between SRL and ECA. The meta-analytical results showed a z value = .15 (CI [.11, .19]), which was converted into *r* (*r* = .15).

*Self-regulated learning and cognitive abilities*

Only one effect size was retrieved from the literature for the correlation between SRL and cognitive abilities (Zuffianò et al., 2013). It showed a z value = .10 (CI [-.05, .25]), which was converted into *r* (*r* = .10).

*Self-regulated learning and life satisfaction*

Two effect sizes were retrieved from one study for the correlation between SRL and life satisfaction. They yielded a z value = .17 (CI [.10, .25]), which was converted into *r* (*r* = .17).

*Self-regulated learning and academic achievement*

The five meta-analytical effects corresponding to the facets of SRL considered in our study were retrieved from the meta-analysis conducted by Richardson et al. (2013). In particular, organization (z = .20, CI for *r* [.09, .20]), elaboration (z = .14, CI for *r* [.03, .25]), metacognition (z = .14, CI for *r* [.05, .22]), critical thinking (z = .16, CI for *r* [.16, .16]), and study management (z = .20, CI for *r* [.17, .20]) showed positive relations with academic achievement. The average of these effects was .17 (*r* = .17).

*Motivation and positive emotions*

We retrieved 45 effect sizes for the correlations between the three aspects of motivation and PA: 12 for self-efficacy (from five studies), 25 for learning goals (from the meta-analysis by Huang et al., 2011), and 8 for incremental theories of intelligence (from three studies). In particular, PA were positively related with self-efficacy (z = .41, CI [.30, .53]), learning goals (z = .43, CI for *r* [.35, .47]), and incremental theories of intelligence (z = .09, CI [-.01, .20]). The mean of the three values obtained was computed (mean z = .33) and converted into *r* (*r* = .32).

*Motivation and negative emotions*

We retrieved 50 effect sizes for the correlations between the three aspects of motivation and NA: 15 for self-efficacy (from five studies), 25 for learning goals (from the meta-analysis by Huang et al., 2011), and 10 for incremental theories of intelligence (from three studies). In particular, NA related with self-efficacy (z = -.38, CI [-.48, -.29]), learning goals (z = .13, CI for *r* [.07, .19]), and incremental theories of intelligence (z = -.21, CI [-.39, -.11]). The mean of the three values obtained was computed (mean z = -.12) and converted into *r* (*r* = -.12).

*Motivation and extracurricular activities*

We retrieved 4 effect sizes for the correlations between the three aspects of motivation and ECA: 2 for self-efficacy (from two studies), 2 for learning goals, and 2 for incremental theories of intelligence. In particular, ECA related with self-efficacy (z = .16, CI [.01, .32]), learning goals (z = .02, CI [-.05, .08]), and incremental theories of intelligence (z = .02, CI [-.05, .09]). The mean of the three values obtained was computed (mean z = .07) and converted into *r* (*r* = .07).

*Motivation and cognitive abilities*

The meta-analysis by Kriegbaum et al. (2018) was used for the prior for the relation between cognitive abilities and academic achievement. This value was *r* = .17 (CI [.15, .20]).

*Motivation and life satisfaction*

We retrieved 9 effect sizes for the correlations between the three aspects of motivation and life satisfaction: 7 for self-efficacy (from four studies), 2 for learning goals (from one study), and none for incremental theories of intelligence. In particular, cognitive abilities positively related with self-efficacy (z = .30, CI [.28, .33]),and learning goals (z = .38, CI [.28, .48]). The mean of the values obtained was computed (mean z = .34) and converted into *r* (*r* = .33).

*Motivation and academic achievement*

The 221 effect sizes retrieved from three different meta-analyses showed that academic achievement is positively related to self-efficacy (z = .29, CI for *r* [.14, .41]; Richardson et al., 2012), learning goals (z = .12, CI for *r* [.03, .21]; Richardson et al., 2012), and incremental theories of intelligence (z = .07, CI [.04, .11]; Costa & Faria, 2018). The mean of the three values obtained was computed (mean z = .19) and converted into *r* (*r* = .19).

*Positive emotions and negative emotions*

For the prior on the correlation between positive and negative emotions, we used the results of the validation of the PANAS for Italian youth by Ciucci et al., 2017. This resulted in a value of -.28.

*Positive emotions and extracurricular activities*

Three effect sizes were retrieved from two studies for the correlation between PA and ECA. The meta-analytical results showed a z value = .20 (CI [.12, .29]) that was converted into *r* (*r* = .20).

*Positive emotions and cognitive abilities*

Only one effect size was retrieved (from Zaccoletti et al., 2020) for the correlation between PA and cognitive abilities. In this study, *r* was .12.

*Positive emotions and life satisfaction*

Ten effect sizes were retrieved from seven studies for the correlation between PA and life satisfaction. The meta-analytical results showed a z value = .42 (CI [.39, .45]), that was converted into *r* (*r* = .40).

*Positive emotions and academic achievement*

Nine effect sizes were retrieved from five studies for the correlation between PA and academic achievement. The meta-analytical results showed a z value = .12 (CI [.09, .16]) that was converted into *r* (*r* = .12).

*Negative emotions and extracurricular activities*

Two effect sizes were retrieved from two studies for the correlation between NA and ECA. The meta-analytical results showed a z value = -.12 (CI [-.17, -.07]), that was converted into *r* (*r* = -.12).

*Negative emotions and cognitive abilities*

Two effect sizes were retrieved (from Zaccoletti et al., 2020) for the correlation between NA and cognitive abilities. The meta-analytical results showed a z value = .09 (CI [-.41, .23]), that was converted into *r* (*r* = .09).

*Negative emotions and life satisfaction*

Ten effect sizes were retrieved from seven studies for the correlation between NA and life satisfaction. The meta-analytical results showed a z value = -.36 (CI [-.45, -.26]), that was converted into *r* (*r* = -.35).

*Negative emotions and academic success*

Thirteen effect sizes were retrieved from six studies for the correlation between NA and academic success. The meta-analytical results showed a z value = -.16 (CI [-.22, -.11]), that was converted into *r* (*r* = -.16).

*Extracurricular activities and cognitive abilities*

Two effect sizes were retrieved (from Feraco et al., 2020) for the correlation between ECA and cognitive abilities. The meta-analytical results showed a z value = .02 (CI [-.05, .09]), that was converted into *r* (*r* = .02).

*Extracurricular activities and life satisfaction*

Two effect sizes were retrieved from two studies for the correlation between ECA and life satisfaction. The meta-analytical results showed a z value = .17 (CI [.13, .22]), that was converted into *r* (*r* = .17).

*Extracurricular activities and academic achievement*

The value in the meta-analysis by Shulruf (2010) was used as the prior for the relation between ECA and academic achievement. This value was r = .13 (CI [-.15, 31]).

*Cognitive abilities and life satisfaction*

The literature review by Suldo et al., (2006) was used as an indication of the relation between cognitive abilities and life satisfaction. The authors found no consistency in the literature, so we set this value as *N*(.10, .20).

*Cognitive abilities and academic achievement*

The meta-analysis by Roth et al. (2015) was used for the prior for the relation between cognitive abilities and academic achievement. This value was *r* = .54 (CI [.51, .57]).

*Life satisfaction and academic achievement*

Seven effect sizes were retrieved from six studies for the correlation between academic achievement and life satisfaction. The meta-analytical results showed a z value = .23 (CI [.19, .26]), which was converted into *r* (*r* = .23).

**Supplementary tables**

**Table S1.**

Means, standard deviations, omega, and correlations between all study variables.

|  | M | SD | ω | 1. | 2. | 3. | 4. | 5. | 6. | 7. | 8. | 9. | 10. | 11. | 12. | 13. | 14. |
| --- | --- | --- | --- | --- | --- | --- | --- | --- | --- | --- | --- | --- | --- | --- | --- | --- | --- |
| 1.Adaptability | 4.04 | 0.78 | .72 | 1 |  |  |  |  |  |  |  |  |  |  |  |  |  |
| 2.Curiosity | 4.33 | 0.76 | .79 | .36* | 1 |  |  |  |  |  |  |  |  |  |  |  |  |
| 3.Perseverance | 4.50 | 0.71 | .63 | .38* | .55* | 1 |  |  |  |  |  |  |  |  |  |  |  |
| 4.Initiative | 4.20 | 0.79 | .65 | .47* | .49* | .57* | 1 |  |  |  |  |  |  |  |  |  |  |
| 5.Social awareness | 4.77 | 0.77 | .74 | .21* | .55* | .38* | .33* | 1 |  |  |  |  |  |  |  |  |  |
| 6.Leadership | 4.10 | 0.99 | .71 | .36* | .33* | .50* | .68* | .22* | 1 |  |  |  |  |  |  |  |  |
| 7.Soft skills | 4.32 | 0.58 | .88 | .64* | .74* | .77* | .83* | .60* | .75* | 1 |  |  |  |  |  |  |  |
| 8.SRL | 3.37 | 0.39 | .72 | .23* | .57* | .53* | .41* | .46* | .29* | .56* | 1 |  |  |  |  |  |  |
| 9.Motivation | 3.54 | 0.52 | .71 | .24* | .51* | .44* | .28* | .34* | .23* | .46* | .51* | 1 |  |  |  |  |  |
| 10.PA | 3.17 | 0.72 | .77 | .30* | .51* | .49* | .44* | .28* | .40* | .56* | .52* | .36* | 1 |  |  |  |  |
| 11.NA | 2.25 | 0.82 | .88 | -.32* | -.13 | -.19* | -.14* | .07 | -.14* | -.20* | -.15* | -.19* | -.15* | 1 |  |  |  |
| 12.ECA | 8.62 | 3.16 | - | .04 | .10 | .13 | .17* | .12 | .16* | .17* | .15* | .08 | .08 | -.01 | 1 |  |  |
| 13.Cognitive abilities | 32.10 | 5.07 | .77^a^ | .10 | .08 | .12 | .14* | .06 | .21* | .17* | .10 | .11 | .02 | -.11 | .17* | 1 |  |
| 14.Life satisfaction | 4.62 | 1.28 | .83 | .24* | .29* | .38* | .31* | .12 | .30* | .38* | .30* | .25* | .43* | -.25* | .11 | .05 | 1 |
| 15.Academic achievement | 7.10 | 1.09 | .69 | .02 | .19* | .26* | .15* | .17* | .13 | .21* | .32* | .34* | .17* | -.17* | .20* | .35* | .17* |

* p < .001.

^a^The α for the Culture-Free Intelligence Test refers to split-half reliability

Note. SRL = Self-regulated learning; PA = Positive emotions; NA = Negative emotions; ECA = Extracurricular activities

**Table S2.** List of the studies considered for the purpose of specifying the priors, and related effects.

| Study | N^a^ | Relation | r | z | CI | Second-order  relation | r |
| --- | --- | --- | --- | --- | --- | --- | --- |
| 1. Martin et al., 2013 | 969 | AD → SRL | .54 | .44 | [.35, .54] | SS → SRL | .39 |
| 1. Feraco et al., 2021 | 448 | AD → SRL | .24 |  |  |  |  |
| 1. Chan et al., 2012 | 1381 | CU → SRL | .48 | .40 | [.22, .58] |  |  |
| 1. Chan et al., 2012 | 1381 | CU → SRL | .01 |  |  |  |  |
| 1. Richards et al., 2013 | 90 | CU → SRL | .54 |  |  |  |  |
| 1. Richards et al., 2013 | 90 | CU → SRL | .52 |  |  |  |  |
| 1. Pekrun et al., 2016 | 438 | CU → SRL | .30 |  |  |  |  |
| 1. Feraco et al., 2021 | 448 | CU → SRL | .42 |  |  |  |  |
| 1. Feraco et al., 2021 | 448 | IN → SRL | .42 | .44 | [.35, .54] |  |  |
| 1. Feraco et al., 2021 | 448 | PE → SRL | .30 | .31 | [22, .40] |  |  |
| 1. Weisskirch, 2018 | 3023 | PE → SRL | .51 |  |  |  |  |
| 1. Weisskirch, 2018 | 302 | PE → SRL | .38 |  |  |  |  |
| 1. Weisskirch, 2018 | 302 | PE → SRL | .38 |  |  |  |  |
| 1. Wolters & Hussain, 2015 | 213 | PE → SRL | .50 |  |  |  |  |
| 1. Feraco et al., 2021 | 448 | PE → SRL | .50 |  |  |  |  |
| 1. Feraco et al., 2021 | 448 | SA → SRL | .38 | .50 | [.43, .60] |  |  |
| 1. Feraco et al., 2021 | 448 | AD → IT | .01 | .24 | [.07, .41] | SS → SM | .26 |
| 1. Martin et al., 2013 | 969 | AD → IT | .25 |  |  |  |  |
| 1. Martin et al., 2012 | 2731 | AD → IT | .25 |  |  |  |  |
| 1. Martin et al., 2012 | 2731 | AD → IT | .41 |  |  |  |  |
| 1. Feraco et al., 2021 | 448 | CU → IT | .12 | .12 | [.03, .21] |  |  |
| 1. Feraco et al., 2021 | 448 | IN → IT | .17 | .17 | [.08, .26] |  |  |
| 1. Feraco et al., 2021 | 448 | LE → IT | .01 | .01 | [-.08, .10] |  |  |
| 1. Burgoyne et al., 2018 | 488 | PE → IT | .15 | .23 | [.13, .34] |  |  |
| 1. Dixson et al., 2017 | 105 | PE → IT | .39 |  |  |  |  |
| 1. Feraco et al., 2021 | 448 | PE → IT | .05 |  |  |  |  |
| 1. Myers et al., 2016 | 9 | PE → IT | .34 |  |  |  |  |
| 1. Karlen et al., 2019 | 1215 | PE → IT | .13 |  |  |  |  |
| 1. Park et al., 2020 | 1667 | PE → IT | .20 |  |  |  |  |
| 1. Sigmundsson et al., 2020 | 146 | PE → IT | .27 |  |  |  |  |
| 1. Tang et al., 2019 | 2018 | PE → IT | .15 |  |  |  |  |
| 1. West et al., 2016 | 1368 | PE → IT | .18 |  |  |  |  |
| 1. Zhao et al, 2018 | 1842 | PE → IT | .51 |  |  |  |  |
| 1. Feraco et al., 2021 | 448 | SA → IT | -.01 | -.01 | [-.10, .08] |  |  |
| 1. Burns et al., 2018 | 1481 | AD → SE | .70 | .47 | [.29, .67] |  |  |
| 1. Burns et al., 2018 | 1481 | AD → SE | .29 |  |  |  |  |
| 1. Feraco et al., 2021 | 448 | AD → SE | .24 |  |  |  |  |
| 1. Feraco et al., 2021 | 448 | AD → SE | .15 |  |  |  |  |
| 1. Martin et al., 2016 | 3617 | AD → SE | .56 |  |  |  |  |
| 1. Martin et al., 2016 | 989 | AD → SE | .51 |  |  |  |  |
| 1. Martin et al., 2016 | 1182 | AD → SE | .52 |  |  |  |  |
| 1. Anderson et al., 2019 | 447 | CU → SE | .33 | .38 | [.33, .44] |  |  |
| 1. Feraco et al., 2021 | 448 | CU → SE | .36 |  |  |  |  |
| 1. Feraco et al., 2021 | 448 | CU → SE | .32 |  |  |  |  |
| 1. Ruch et al., 2014 | 2110 | CU → SE | .41 |  |  |  |  |
| 1. Fay & Frese, 2001 | 152 | IN → SE | .24 | .24 | [.16, .33] |  |  |
| 1. Fay & Frese, 2001 | 152 | IN → SE | .25 |  |  |  |  |
| 1. Fay & Frese, 2001 | 152 | IN → SE | .08 |  |  |  |  |
| 1. Fay & Frese, 2001 | 152 | IN → SE | .21 |  |  |  |  |
| 1. Feraco et al., 2021 | 448 | IN → SE | .22 |  |  |  |  |
| 1. Feraco et al., 2021 | 448 | IN → SE | .37 |  |  |  |  |
| 1. Feraco et al., 2021 | 448 | LE → SE | .16 | .30 | [.10, .49] |  |  |
| 1. Feraco et al., 2021 | 448 | LE → SE | .23 |  |  |  |  |
| 1. Ruch et al., 2014 | 2110 | LE → SE | .45 |  |  |  |  |
| 1. Credé et al., 2017 | M | PE → SE |  | .46 | [.28, .57] |  |  |
| 1. Feraco et al., 2021 | 448 | SA → SE | .07 | .32 | [-.16, .80] |  |  |
| 1. Ruch et al., 2014 | 2110 | SA → SE | .51 |  |  |  |  |
| 1. Feraco et al., 2021 | 448 | AD → LG | .14 | .39 | [.25, .53] |  |  |
| 1. Holliman et al., 2018 | 90 | AD → LG | .32 |  |  |  |  |
| 1. Martin et al., 2016 | 3617 | AD → LG | .49 |  |  |  |  |
| 1. Martin et al., 2016 | 989 | AD → LG | .44 |  |  |  |  |
| 1. Martin et al., 2016 | 1182 | AD → LG | .40 |  |  |  |  |
| 1. Chan et al., 2012 | 1381 | CU → LG | .45 | .47 | [.43, 52] |  |  |
| 1. Feraco et al., 2021 | 448 | CU → LG | .41 |  |  |  |  |
| 1. Richards et al, 2013 | 90 | CU → LG | .47 |  |  |  |  |
| 1. Feraco et al., 2021 | 448 | IN → LG | .22 | .22 | [.13, .22] |  |  |
| 1. Feraco et al., 2021 | 448 | LE → LG | .08 | .18 | [-.03, .40] |  |  |
| 1. Weber et al., 2016 | 196 | LE → LG | .29 |  |  |  |  |
| 1. Feraco et al., 2021 | 448 | PE → LG | .31 | .38 | [.29, .47] |  |  |
| 1. Karlen et al., 2019 | 1215 | PE → LG | .25 |  |  |  |  |
| 1. Karlen et al., 2019 | 1215 | PE → LG | .40 |  |  |  |  |
| 1. Muenks et al., 2018 | 190 | PE → LG | .38 |  |  |  |  |
| 1. Muenks et al., 2018 | 190 | PE → LG | .22 |  |  |  |  |
| 1. Zhao et al, 2018 | 1842 | PE → LG | .50 |  |  |  |  |
| 1. Feraco et al., 2021 | 448 | SA → LG | .26 | .28 | [.20, .36] |  |  |
| 1. Weber et al., 2016 | 196 | SA → LG | .31 |  |  |  |  |
| 1. Austin et al., 2010 | 475 | AD → PA | .19 | .21 | [.14, .29] | SS → PA | .38 |
| 1. Saklofske et al., 2012 | 238 | AD → PA | .25 |  |  |  |  |
| 1. Anderson et al., 2020 | 447 | CU → PA | .36 | .42 | [.38, .47] |  |  |
| 1. Anderson et al., 2020 | 447 | CU → PA | .40 |  |  |  |  |
| 1. Dametto & Noronha, 2019 | 826 | CU → PA | .40 |  |  |  |  |
| 1. Gallagher & Lopez, 2007 | 293 | CU → PA | .50 |  |  |  |  |
| 1. Güsewell & Ruch, 2012 | 574 | CU → PA | .36 |  |  |  |  |
| 1. Kashdan et al., 2009 | 150 | CU → PA | .28 |  |  |  |  |
| 1. Kashdan et al., 2004 | 514 | CU → PA | .44 |  |  |  |  |
| 1. Macaskill & Denovan, 2014 | 214 | CU → PA | .50 |  |  |  |  |
| 1. Neff et al., 2006 | 177 | CU → PA | .37 |  |  |  |  |
| 1. Ros-Morente et al., 2018 | 419 | CU → PA | .33 |  |  |  |  |
| 1. Weber et al., 2016 | 196 | CU → PA | .46 |  |  |  |  |
| 1. Robitschek & Keyes, 2009 | 467 | IN → PA | .38 | .40 | [.31, .49] |  |  |
| 1. Dametto & Noronha, 2019 | 826 | LE → PA | .35 | .38 | [.31, .45] |  |  |
| 1. Ros-Morente et al., 2018 | 419 | LE → PA | .32 |  |  |  |  |
| 1. Weber et al., 2016 | 196 | LE → PA | .46 |  |  |  |  |
| 1. Dametto & Noronha, 2019 | 826 | PE → PA | .42 | .50 | [.40, .60] |  |  |
| 1. Datu et al., 2019 | 606 | PE → PA | .43 |  |  |  |  |
| 1. Datu et al., 2019 | 220 | PE → PA | .49 |  |  |  |  |
| 1. Datu & Fong, 2018 | 1051 | PE → PA | .56 |  |  |  |  |
| 1. Datu & Fong, 2018 | 1051 | PE → PA | .35 |  |  |  |  |
| 1. Ros-Morente et al., 2018 | 419 | PE → PA | .35 |  |  |  |  |
| 1. Weber et al., 2016 | 196 | PE → PA | .63 |  |  |  |  |
| 1. Dametto & Noronha, 2019 | 826 | SA → PA | .41 | .46 | [.30, .62] |  |  |
| 1. Ros-Morente et al., 2018 | 419 | SA → PA | .32 |  |  |  |  |
| 1. Weber et al., 2016 | 196 | SA → PA | .56 |  |  |  |  |
| 1. Austin et al., 2010 | 475 | AD → NA | -.12 | -.16 | [-.26, -.06] | SS → NA | -.19 |
| 1. Saklofske et al., 2012 | 238 | AD → NA | -.22 |  |  |  |  |
| 1. Dametto & Noronha, 2019 | 826 | CU → NA | -.11 | -14 | [-.21, -.07] |  |  |
| 1. Gallagher & Lopez, 2007 | 293 | CU → NA | -.28 |  |  |  |  |
| 1. Kashdan et al., 2009 | 150 | CU → NA | -.05 |  |  |  |  |
| 1. Kashdan et al., 2004 | 514 | CU → NA | -.22 |  |  |  |  |
| 1. Macaskill & Denovan, 2014 | 214 | CU → NA | -.17 |  |  |  |  |
| 1. Neff et al., 2007 | 177 | CU → NA | -.08 |  |  |  |  |
| 1. Weber et al., 2016 | 196 | CU → NA | -.02 |  |  |  |  |
| 1. Weigold et al., 2020 | M | IN → NA |  | -.25 | [-.39, -.01] |  |  |
| 1. Dametto & Noronha, 2019 | 826 | LE → NA | -.15 | -.16 | [-.23, -.10] |  |  |
| 1. Weber et al., 2016 | 196 | LE → NA | -.22 |  |  |  |  |
| 1. Dametto & Noronha, 2019 | 826 | PE → NA | -.27 | -.18 | [-.25, -.11] |  |  |
| 1. Datu et al., 2016 | 606 | PE → NA | -.13 |  |  |  |  |
| 1. Datu et al., 2016 | 220 | PE → NA | -.26 |  |  |  |  |
| 1. Datu et al., 2018 | 447 | PE → NA | -.21 |  |  |  |  |
| 1. Datu & Fong, 2018 | 1051 | PE → NA | -.06 |  |  |  |  |
| 1. Datu & Fong, 2018 | 1051 | PE → NA | -.09 |  |  |  |  |
| 1. Weber et al., 2016 | 196 | PE → NA | -.28 |  |  |  |  |
| 1. Dametto & Noronha, 2019 | 826 | SA → NA | -.23 | -.25 | [-.31, -.19] |  |  |
| 1. Weber et al., 2016 | 196 | SA → NA | -.30 |  |  |  |  |
| 1. Feraco et al., 2021 | 448 | AD → ECA | .08 | .08 | [-.01, .17] | SS → ECA | .12 |
| 1. Feraco et al., 2021 | 448 | CU → ECA | .06 | .06 | [-.03, .15] |  |  |
| 1. Feraco et al., 2021 | 448 | IN → ECA | .19 | .19 | [.10, .29] |  |  |
| 1. Feraco et al., 2021 | 448 | LE → ECA | .19 | .19 | [.10, .29] |  |  |
| 1. Feraco et al., 2021 | 448 | PE → ECA | .07 | .15 | [-.02, .33] |  |  |
| 1. Lufi & Tenenbaum, 1991 | 149 | PE → ECA | .25 |  |  |  |  |
| 1. Feraco et al., 2021 | 448 | SA → ECA | .08 | .08 | [-.01, .17] |  |  |
| 1. Derksen et al., 2002 | 873 | AD → CA | .10 | .09 | [.04, .14] | SS → CA | .07 |
| 1. Feraco et al., 2021 | 448 | AD → CA | .07 |  |  |  |  |
| 1. Newsome et al., 2000 | 137 | AD → CA | .09 |  |  |  |  |
| 1. Von Stumm et al., 2011 | M | CU → CA |  | .22 |  |  |  |
| 1. Fay & Frese, 2001 | 152 | IN → CA | .28 | .19 | [.01, .36] |  |  |
| 1. Fay & Frese, 2001 | 152 | IN → CA | .27 |  |  |  |  |
| 1. Feraco et al., 2021 | 448 | IN → CA | .03 |  |  |  |  |
| 1. Feraco et al., 2021 | 448 | LE → CA | -.06 | -.06 | [-.15, .03] |  |  |
| 1. Credé et al., 2001 | M | PE → CA |  | -.01 | [-.06, .04] |  |  |
| 1. Feraco et al., 2021 | 448 | SA → CA | .01 | .01 | [-.08, .10] |  |  |
| 1. Austin et al., 2010 | 475 | AD → LS | .10 | .44 | [.25, .64] | SS → LS | .36 |
| 1. Holliman et al., 2018 | 90 | AD → LS | .44 |  |  |  |  |
| 1. Martin et al., 2013 | 969 | AD → LS | .58 |  |  |  |  |
| 1. Martin et al., 2012 | 2731 | AD → LS | .62 |  |  |  |  |
| 1. Saklofske et al., 2012 | 238 | AD → LS | .23 |  |  |  |  |
| 1. Zhou & Lin, 2016 | 99 | AD → LS | .39 |  |  |  |  |
| 1. Bruna et al., 2019 | M | CU → LS |  | .42 | [.38, .42] |  |  |
| 1. Chang et al, 2019 | 152 | IN → LS | .40 | .53 | [.31, .75] |  |  |
| 1. Robitschek & Keyes, 2009 | 467 | IN → LS | .40 |  |  |  |  |
| 1. Stevic & Ward, 2008 | 204 | IN → LS | .64 |  |  |  |  |
| 1. Bruna et al., 2019 | M | LE → LS |  | .23 | [.20, .29] |  |  |
| 1. Bruna et al., 2019 | M | PE → LS |  | .33 | [.26, .37] |  |  |
| 1. Bruna et al., 2019 | M | SA → LS |  | .30 | [.26, .32] |  |  |
| 1. Austin et al., 2010 | 475 | AD → AA | .24 | .18 | [.10, .26] | SS → AA | .19 |
| 1. Burns et al., 2018 | 1481 | AD → AA | .19 |  |  |  |  |
| 1. Collie et al., 2016 | 186 | AD → AA | .05 |  |  |  |  |
| 1. Martin et al., 2012 | 2731 | AD → AA | .30 |  |  |  |  |
| 1. Martin et al., 2015 | 969 | AD → AA | .16 |  |  |  |  |
| 1. Holliman et al., 2018 | 90 | AD → AA | .35 |  |  |  |  |
| 1. Newsome et al., 2000 | 137 | AD → AA | .08 |  |  |  |  |
| 1. Von Stumm et al., 2011 | M | CU → AA |  | .33 |  |  |  |
| 1. Feraco et al., 2021 | 448 | IN → AA | .09 | .09 | [-.02, .18] |  |  |
| 1. Feraco et al., 2021 | 448 | LE → AA | .12 | .13 | [.06, .19] |  |  |
| 1. Lounsbury et al., 2009 | 237 | LE → AA | .14 |  |  |  |  |
| 1. Wagner & Ruch, 2015 | 378 | LE → AA | .12 |  |  |  |  |
| 1. Credé et al., 2017 | M | PE → AA |  | .27 | [.11, .41] |  |  |
| 1. Feraco et al., 2021 | 448 | SA → AA | .08 | .11 | [.08, .17] |  |  |
| 1. Lounsbury et al., 2009 | 237 | SA → AA | .10 |  |  |  |  |
| 1. Wagner & Ruch, 2015 | 378 | SA → AA | .17 |  |  |  |  |
| 1. Wagner & Ruch, 2015 | 378 | SA → AA | .12 |  |  |  |  |
| 1. Wagner & Ruch, 2015 | 378 | SA → AA | .17 |  |  |  |  |
| 1. Mega et al., 2014 | 5805 | SRL → SE | .49 | .41 | [.35, .47] | SRL → SM | .29 |
| 1. Mega et al., 2014 | 5805 | SRL → SE | .36 |  |  |  |  |
| 1. Mega et al., 2014 | 5805 | SRL → SE | .35 |  |  |  |  |
| 1. Mega et al., 2014 | 5805 | SRL → SE | .39 |  |  |  |  |
| 1. Mega et al., 2014 | 5805 | SRL → SE | .40 |  |  |  |  |
| 1. Wolter & Hussain 2014 | 213 | SRL → SE | .30 |  |  |  |  |
| 1. Mega et al., 2014 | 5805 | SRL → LG | .28 | .25 | [.17, .33] |  |  |
| 1. Mega et al., 2014 | 5805 | SRL → LG | .36 |  |  |  |  |
| 1. Mega et al., 2014 | 5805 | SRL → LG | .16 |  |  |  |  |
| 1. Mega et al., 2014 | 5805 | SRL → LG | .17 |  |  |  |  |
| 1. Mega et al., 2014 | 5805 | SRL → LG | .26 |  |  |  |  |
| 1. Burnette et al.,2012 | M | SRL → IT |  | .13 | [.16, .29] |  |  |
| 1. Artino & Jones, 2012 | 302 | SRL → PA | .53 | .37 | [.33, .41] | SRL → PA | .36 |
| 1. Artino & Jones, 2012 | 302 | SRL → PA | .51 |  |  |  |  |
| 1. Mega et al., 2014 | 5805 | SRL → PA | .37 |  |  |  |  |
| 1. Mega et al., 2014 | 5805 | SRL → PA | .47 |  |  |  |  |
| 1. Mega et al., 2014 | 5805 | SRL → PA | .42 |  |  |  |  |
| 1. Mega et al., 2014 | 5805 | SRL → PA | .31 |  |  |  |  |
| 1. Mega et al., 2014 | 5805 | SRL → PA | .32 |  |  |  |  |
| 1. Mega et al., 2014 | 5805 | SRL → PA | .37 |  |  |  |  |
| 1. Mega et al., 2014 | 5805 | SRL → PA | .24 |  |  |  |  |
| 1. Mega et al., 2014 | 5805 | SRL → PA | .27 |  |  |  |  |
| 1. Mega et al., 2014 | 5805 | SRL → PA | .23 |  |  |  |  |
| 1. Mega et al., 2014 | 5805 | SRL → PA | .33 |  |  |  |  |
| 1. Mega et al., 2014 | 5805 | SRL → PA | .39 |  |  |  |  |
| 1. Mega et al., 2014 | 5805 | SRL → PA | .34 |  |  |  |  |
| 1. Mega et al., 2014 | 5805 | SRL → PA | .33 |  |  |  |  |
| 1. Mega et al., 2014 | 5805 | SRL → PA | .36 |  |  |  |  |
| 1. Mega et al., 2014 | 5805 | SRL → PA | .36 |  |  |  |  |
| 1. Pekrun et al., 2002 | 230 | SRL → PA | .44 |  |  |  |  |
| 1. Pekrun et al., 2002 | 230 | SRL → PA | .33 |  |  |  |  |
| 1. Pekrun et al., 2011 | 389 | SRL → PA | .26 |  |  |  |  |
| 1. Pekrun et al., 2011 | 389 | SRL → PA | .45 |  |  |  |  |
| 1. Pekrun et al., 2011 | 389 | SRL → PA | .43 |  |  |  |  |
| 1. Pekrun et al., 2011 | 389 | SRL → PA | .14 |  |  |  |  |
| 1. Shih, 2011 | 481 | SRL → PA | .56 |  |  |  |  |
| 1. Artino & Jones, 2012 | 302 | SRL → NA | -.37 | -.28 | [-.31, -.26] | SRL → NA | -.27 |
| 1. Artino & Jones, 2012 | 302 | SRL → NA | -.28 |  |  |  |  |
| 1. Artino & Jones, 2012 | 302 | SRL → NA | -.40 |  |  |  |  |
| 1. Artino & Jones, 2012 | 302 | SRL → NA | -.28 |  |  |  |  |
| 1. Mega et al 2014 | 5805 | SRL → NA | -.31 |  |  |  |  |
| 1. Mega et al 2014 | 5805 | SRL → NA | -.41 |  |  |  |  |
| 1. Mega et al 2014 | 5805 | SRL → NA | -.38 |  |  |  |  |
| 1. Mega et al 2014 | 5805 | SRL → NA | -.20 |  |  |  |  |
| 1. Mega et al 2014 | 5805 | SRL → NA | -.25 |  |  |  |  |
| 1. Mega et al 2014 | 5805 | SRL → NA | -.26 |  |  |  |  |
| 1. Mega et al 2014 | 5805 | SRL → NA | -.24 |  |  |  |  |
| 1. Mega et al 2014 | 5805 | SRL → NA | -.30 |  |  |  |  |
| 1. Mega et al 2014 | 5805 | SRL → NA | -.28 |  |  |  |  |
| 1. Mega et al 2014 | 5805 | SRL → NA | -.23 |  |  |  |  |
| 1. Mega et al 2014 | 5805 | SRL → NA | -.3 |  |  |  |  |
| 1. Mega et al 2014 | 5805 | SRL → NA | -.26 |  |  |  |  |
| 1. Mega et al 2014 | 5805 | SRL → NA | -.22 |  |  |  |  |
| 1. Mega et al 2014 | 5805 | SRL → NA | -.28 |  |  |  |  |
| 1. Mega et al 2014 | 5805 | SRL → NA | -.27 |  |  |  |  |
| 1. Pekrun et al.,2002 | 230 | SRL → NA | -.21 |  |  |  |  |
| 1. Pekrun et al.,2002 | 230 | SRL → NA | -.22 |  |  |  |  |
| 1. Pekrun et al.,2002 | 230 | SRL → NA | -.26 |  |  |  |  |
| 1. Pekrun et al.,2011 | 389 | SRL → NA | -.25 |  |  |  |  |
| 1. Pekrun et al.,2011 | 389 | SRL → NA | -.29 |  |  |  |  |
| 1. Pekrun et al.,2011 | 389 | SRL → NA | -.26 |  |  |  |  |
| 1. Pekrun et al.,2011 | 389 | SRL → NA | -.34 |  |  |  |  |
| 1. Pekrun et al.,2011 | 389 | SRL → NA | -.04 |  |  |  |  |
| 1. Shih, 2011 | 481 | SRL → NA | -.20 |  |  |  |  |
| 1. Feraco et al., 2021 | 448 | SRL → ECA | .13 | .15 | [.11, .19] | SRL → ECA | .15 |
| 1. Guilmette 2019 | 401 | SRL → ECA | .21 |  |  |  |  |
| 1. Guilmette 2019 | 401 | SRL → ECA | .11 |  |  |  |  |
| 1. Zuffianò et al., 2013 | 170 | SRL → CA | .10 | .10 | [-.05, .25] | SRL → CA | .10 |
| 1. Antaramian, 2017 | 357 | SRL → LS | .17 | .17 | [.10, .25] | SRL → LS | .17 |
| 1. Antaramian, 2017 | 357 | SRL → LS | .17 |  |  |  |  |
| 1. Richardson et al., 2013 | M | SRL → AA |  | .20 | [.09, .20] | SRL → AA | .17 |
| 1. Richardson et al., 2013 | M | SRL → AA |  | .14 | [.03, .25] |  |  |
| 1. Richardson et al., 2013 | M | SRL → AA |  | .14 | [.05, .22] |  |  |
| 1. Richardson et al., 2013 | M | SRL → AA |  | .16 | [.16, .16] |  |  |
| 1. Richardson et al., 2013 | M | SRL → AA |  | .20 | [.17, .20] |  |  |
| 1. Artino and Jones, 2012 | 302 | SE → PA | .22 | .41 | [.30, .53] | SM → PA | .32 |
| 1. Mega et al 2014 | 5805 | SE → PA | .51 |  |  |  |  |
| 1. Mega et al 2014 | 5805 | SE → PA | .62 |  |  |  |  |
| 1. Mega et al 2014 | 5805 | SE → PA | .54 |  |  |  |  |
| 1. Artino et al.,2010 | 136 | SE → PA | .27 |  |  |  |  |
| 1. Pekrun et al.,2011 | 389 | SE → PA | .37 |  |  |  |  |
| 1. Pekrun et al.,2011 | 389 | SE → PA | .53 |  |  |  |  |
| 1. Pekrun et al.,2011 | 389 | SE → PA | .51 |  |  |  |  |
| 1. Pekrun et al.,2011 | 389 | SE → PA | .07 |  |  |  |  |
| 1. Putwain et al 2012 | 206 | SE → PA | .26 |  |  |  |  |
| 1. Putwain et al 2012 | 206 | SE → PA | .27 |  |  |  |  |
| 1. Putwain et al 2012 | 206 | SE → PA | .32 |  |  |  |  |
| 1. Huang et al., 2011 | M | LG → PA |  | .43 | [.35, .47] |  |  |
| 1. King et al.,2012 | 1147 | IT → PA | -.06 | .09 | [-.01, .20] |  |  |
| 1. King et al.,2012 | 1147 | IT → PA | -.06 |  |  |  |  |
| 1. King et al.,2012 | 1147 | IT → PA | -.04 |  |  |  |  |
| 1. King, 2012 | 676 | IT → PA | .11 |  |  |  |  |
| 1. Mega et al 2014 | 5805 | IT → PA | .22 |  |  |  |  |
| 1. Mega et al 2014 | 5805 | IT → PA | .19 |  |  |  |  |
| 1. Mega et al 2014 | 5805 | IT → PA | .25 |  |  |  |  |
| 1. Shih, 2011 | 481 | IT → PA | .39 |  |  |  |  |
| 1. Artino et al.,2010 | 136 | SE → NA | -.36 | -.38 | [-.48, -.29] | SM → NA | -.12 |
| 1. Artino et al.,2010 | 136 | SE → NA | -.24 |  |  |  |  |
| 1. Artino & Jones, 2012 | 302 | SE → NA | -.21 |  |  |  |  |
| 1. Artino & Jones, 2012 | 302 | SE → NA | -.10 |  |  |  |  |
| 1. Mega et al 2014 | 5805 | SE → NA | -.39 |  |  |  |  |
| 1. Mega et al 2014 | 5805 | SE → NA | -.54 |  |  |  |  |
| 1. Mega et al 2014 | 5805 | SE → NA | -.48 |  |  |  |  |
| 1. Pekrun et al.,2011 | 389 | SE → NA | -.35 |  |  |  |  |
| 1. Pekrun et al.,2011 | 389 | SE → NA | -.39 |  |  |  |  |
| 1. Pekrun et al.,2011 | 389 | SE → NA | -.48 |  |  |  |  |
| 1. Pekrun et al.,2011 | 389 | SE → NA | -.67 |  |  |  |  |
| 1. Pekrun et al.,2011 | 389 | SE → NA | -.29 |  |  |  |  |
| 1. Putwain et al 2012 | 206 | SE → NA | -.14 |  |  |  |  |
| 1. Putwain et al 2012 | 206 | SE → NA | -.30 |  |  |  |  |
| 1. Putwain et al 2012 | 206 | SE → NA | -.31 |  |  |  |  |
| 1. Huang et al., 2011 | M | LG → NA |  | .13 | [.07, .19] |  |  |
| 1. King, 2012 | 676 | IT → NA | -.06 | -.21 | [-.39, -.11] |  |  |
| 1. King et al.,2012 | 1147 | IT → NA | -.27 |  |  |  |  |
| 1. King et al.,2012 | 1147 | IT → NA | -.26 |  |  |  |  |
| 1. King et al.,2012 | 1147 | IT → NA | -.31 |  |  |  |  |
| 1. King et al.,2012 | 1147 | IT → NA | -.34 |  |  |  |  |
| 1. King et al.,2012 | 1147 | IT → NA | -.40 |  |  |  |  |
| 1. Mega et al 2014 | 5805 | IT → NA | -.05 |  |  |  |  |
| 1. Mega et al 2014 | 5805 | IT → NA | -.04 |  |  |  |  |
| 1. Mega et al 2014 | 5805 | IT → NA | -.08 |  |  |  |  |
| 1. Shih, 2011 | 481 | IT → NA | -.19 |  |  |  |  |
| 1. Feraco et al., 2021 | 448 | SE → ECA | .02 | .16 | [.01, .32] | SM → ECA | .07 |
| 1. Marsh, 1992 | 10613 | SE → ECA | .29 |  |  |  |  |
| 1. Feraco et al., 2021 | 448 | LG → ECA | .02 | .02 | [-.05, .08] |  |  |
| 1. Feraco et al., 2021 | 448 | IT → ECA | .02 | .02 | [-.05, .09] |  |  |
| 1. Kriegbaum et al., 2018 | M | SM → CA |  | .17 | [.15, .20] | SM → CA | .17 |
| 1. Antaramian 2017 | 357 | SE → LS | .25 | .30 | [.28, .33] | SM → LS | .33 |
| 1. Danielsen et al., 2009 | 3358 | SE → LS | .30 |  |  |  |  |
| 1. Diseth et al., 2012 | 240 | SE → LS | .30 |  |  |  |  |
| 1. Huebner & McCollough, 2000 | 92 | SE → LS | .28 |  |  |  |  |
| 1. Diseth et al., 2012 | 240 | LG → LS | .44 | .38 | [.28, .48] |  |  |
| 1. Diseth et al., 2012 | 240 | LG → LS | .28 |  |  |  |  |
| 1. Richardson et al., 2012 | M | SE → AA |  | .29 | [.14, .41] | SM → AA | .19 |
| 1. Richardson et al., 2012 | M | LG → AA |  | .12 | [.03, .21] |  |  |
| 1. Costa & Faria, 2018 | M | IT → AA |  | .07 | [.04, .11] |  |  |
| 1. Gilman & Huebner, 2006 | 485 | PA → ECA | .22 | .20 | [.12, .29] | PA → ECA | .20 |
| 1. Gilman & Huebner, 2006 | 485 | PA → ECA | .08 |  |  |  |  |
| 1. Guilmette et al., 2019 | 401 | PA → ECA | .30 |  |  |  |  |
| 1. Zaccoletti et al.,2020 | 152 | PA → CA | .12 | .12 | [-.04, .28] | PA → CA | .12 |
| 1. Austin et al., 2010 | 475 | PA → LS | .40 | .42 | [.39, .45] | PA → LS | .40 |
| 1. Cohn et al.,2009 | 86 | PA → LS | .32 |  |  |  |  |
| 1. Effner & Antaramian, 2016 | 814 | PA → LS | .40 |  |  |  |  |
| 1. Hagenauer et al., 2017 | 792 | PA → LS | .36 |  |  |  |  |
| 1. Lewis et al.,2009 | 239 | PA → LS | .46 |  |  |  |  |
| 1. Karaztias et al., 2002 | 425 | PA → LS | .49 |  |  |  |  |
| 1. Saklofske et al 2012 | 238 | PA → LS | .40 |  |  |  |  |
| 1. Austin et al., 2010 | 475 | PA → AA | .12 | .12 | [.09, .16] | PA → AA | .12 |
| 1. Daniels et al.,2009 | 669 | PA → AA | .09 |  |  |  |  |
| 1. Daniels et al.,2009 | 669 | PA → AA | .22 |  |  |  |  |
| 1. Daniels et al.,2009 | 669 | PA → AA | .07 |  |  |  |  |
| 1. Guilmette et al., 2019 | 401 | PA → AA | .07 |  |  |  |  |
| 1. Lewis et al.,2009 | 239 | PA → AA | .13 |  |  |  |  |
| 1. Mega et al 2014 | 5805 | PA → AA | .10 |  |  |  |  |
| 1. Mega et al 2014 | 5805 | PA → AA | .19 |  |  |  |  |
| 1. Mega et al 2014 | 5805 | PA → AA | .10 |  |  |  |  |
| 1. Gilman & Huebner 2006 | 485 | NA → ECA | -.12 | -.12 | [-.17, -.07] | NA → ECA | -.12 |
| 1. Guilmette et al., 2019 | 401 | NA → ECA | -.12 |  |  |  |  |
| 1. Zaccoletti et al.,2020 | 152 | NA → CA | -.25 | .09 | [-.41, .23] | NA → CA | .09 |
| 1. Zaccoletti et al.,2020 | 152 | NA → CA | .07 |  |  |  |  |
| 1. Austin et al., 2010 | 475 | NA → LS | -.40 | -.36 | [-.45, -.26] | NA → LS | -.35 |
| 1. Cohn et al., 2009 | 86 | NA → LS | -.16 |  |  |  |  |
| 1. Hagenauer, et al., 2017 | 792 | NA → LS | -.38 |  |  |  |  |
| 1. Effner & Antaramian, 2016 | 814 | NA → LS | -.45 |  |  |  |  |
| 1. Karatzias et al., 2002 | 425 | NA → LS | -.32 |  |  |  |  |
| 1. Lewis et al.,2009 | 239 | NA → LS | -.05 |  |  |  |  |
| 1. Saklofske et al., 2012 | 238 | NA → LS | -.49 |  |  |  |  |
| 1. Austin et al., 2010 | 475 | NA → AA | -.12 | -.16 | [-.22, -.11] | NA → AA | .16 |
| 1. Daniels et al.,2009 | 669 | NA → AA | -.18 |  |  |  |  |
| 1. Daniels et al.,2009 | 669 | NA → AA | -.18 |  |  |  |  |
| 1. Daniels et al.,2009 | 669 | NA → AA | -.38 |  |  |  |  |
| 1. Daniels et al.,2009 | 669 | NA → AA | -.17 |  |  |  |  |
| 1. Daniels et al.,2009 | 669 | NA → AA | -.22 |  |  |  |  |
| 1. Daniels et al.,2009 | 669 | NA → AA | -.15 |  |  |  |  |
| 1. Effner & Antaramian, 2016 | 814 | NA → AA | -.15 |  |  |  |  |
| 1. Guilmette et al., 2019 | 401 | NA → AA | .09 |  |  |  |  |
| 1. Lewis et al.,2009 | 239 | NA → AA | -.18 |  |  |  |  |
| 1. Mega et al 2014 | 5805 | NA → AA | -.11 |  |  |  |  |
| 1. Mega et al 2014 | 5805 | NA → AA | -.22 |  |  |  |  |
| 1. Mega et al 2014 | 5805 | NA → AA | -.14 |  |  |  |  |
| 1. Feraco et al., 2021 | 448 | ECA → CA | .02 | .02 | [-.05, .09] | ECA → CA | .02 |
| 1. Gilman & Huebner, 2006 | 485 | ECA → LS | .17 | .17 | [.13, .22] | ECA → LS | .17 |
| 1. Gilman, 2001 | 321 | ECA → LS | .18 |  |  |  |  |
| 1. Shulruf, 2010 | M | ECA → AA |  | .13 | [-.15, .31] | ECA → AA | .13 |
| 1. Roth et al., 2015 | M | CA → AA |  | .54 | [.51, .57] | CA → AA | .54 |
| 1. Antaramian, 2017 | 357 | LS → CA | .11 | .23 | [.19, .26] | LS → AA | .23 |
| 1. Diseth et al., 2012 | 240 | LS → CA | .26 |  |  |  |  |
| 1. Effner & Antaramian, 2016 | 814 | LS → CA | .21 |  |  |  |  |
| 1. Gilman & Huebner 2006 | 485 | LS → CA | .32 |  |  |  |  |
| 1. Marques et al., 2015 | 682 | LS → CA | .24 |  |  |  |  |
| 1. Suldo & Shaffer, 2008 | 321 | LS → CA | .19 |  |  |  |  |
| 1. Suldo & Shaffer, 2008 | 321 | LS → CA | .21 |  |  |  |  |

^a^ in case a meta-analysis was considered, we referred to it with M

Note. AD = Adaptability; CU = Curiosity; IN = Initiative; LE = Leadership; PE = Perseverance; SA = Social awareness; AA = Academic achievement; LS = Life satisfaction; SRL = Self-regulated learning; SM = Scholastic motivation; PA = Positive emotions; NA = Negative emotions; CA = Cognitive abilities; SS = soft skills; ECA = Extracurricular activities; IT = Intelligence theories; LG = Learning goals; SE = Self-efficacy

**Table S3.** Estimated indirect effects of the two models and associated higher posterior density intervals

|  | Model 1 | | Model 2 | |
| --- | --- | --- | --- | --- |
| Path | β | HPDI 95% | β | HPDI 95% |
| PA→SRL→AA | .04 | [.01, .07] | .06 | [.03, .09] |
| NA→SRL→AA | -.01 | [-.02, .00] | -.01 | [-.02, .01] |
| CA→SRL→AA | .00 | [-.01, .01] | .00 | [-.01, .02] |
| SS→SRL→AA | .05 | [.02, .08] | .07 | [.04, .11] |
| ECA→SRL→AA | .01 | [.00, .02] | .01 | [-.00, .02] |
| PA→SM→AA | .03 | [.01, .05] | .03 | [.01, .06] |
| NA→SM→AA | -.02 | [-.03, -.00] | -.02 | [-.04, -.00] |
| CA→SM→AA | .01 | [-.01, .02] | .01 | [-.01, .03] |
| SS→SM→AA | .06 | [.03, .09] | .07 | [.04, .10] |
| ECA→SM→AA | .01 | [-.00, .02] | -.00 | [-.01, .01] |
| CA→PA→AA | .00 | [-.01, .00] | .00 | [-.01, .01] |
| SS→PA→AA | .02 | [-.02, .05] | .00 | [-.04, .04] |
| ECA→PA→AA | .00 | [.00, .01] | .00 | [.00, .00] |
| CA→NA→AA | .01 | [.00, .02] | .00 | [.00, .01] |
| SS→NA→AA | .02 | [.01, .03] | .01 | [.00, .02] |
| ECA→NA→AA | .00 | [.00, .01] | .00 | [-.01, .00] |
| ECA→CA→AA | .05 | [.02, .07] | .05 | [.03, .08] |
| ECA→SS→AA | .00 | [-.01, .02] | -.01 | [-.02, .00] |
| PA→SRL→LS | .00 | [-.03, .03] | .00 | [-.03, .03] |
| NA→SRL→LS | .00 | [-.01, .00] | .00 | [-.01, .00] |
| CA→SRL→LS | .00 | [-.00, .00] | .00 | [.00, .00] |
| SS→SRL→LS | .00 | [-.03, .03] | .00 | [-.03, .04] |
| ECA→SRL→LS | .00 | [-.01, .01] | .00 | [.00, .01] |
| PA→SM→LS | .01 | [-.01, .02] | .01 | [-.01, .02] |
| NA→SM→LS | .00 | [-.01, .00] | .00 | [-.01, .00] |
| CA→SM→LS | .00 | [.00, .01] | .00 | [-.00, .01] |
| SS→SM→LS | .02 | [-.01, .04] | .02 | [-.01, .04] |
| ECA→SM→LS | .00 | [.00, .01] | .00 | [.00, .00] |
| CA→PA→LS | -.03 | [-.05, -.01] | -.02 | [-.04, .00] |
| SS→PA→LS | .16 | [.11, .21] | .16 | [.11, .21] |
| ECA→PA→LS | .04 | [.02, .06] | .00 | [-.02, .02] |
| CA→NA→LS | .01 | [.00, .03] | .01 | [.00, .03] |
| SS→NA→LS | .03 | [.01, .05] | .03 | [.01, .05] |
| ECA→NA→LS | .01 | [-.01, 02] | .00 | [-.02, .01] |
| ECA→CA→LS | .00 | [-.02, .01] | .00 | [-.02, .01] |
| ECA→SS→LS | .03 | [.01, .05] | .03 | [.01, .04] |
| CA→PA→SRL | -.03 | [-.05, -.01] | -.02 | [-.04, .00] |
| SS→PA→SRL | .16 | [.12, .20] | .17 | [.12, .21] |
| ECA→PA→SRL | .04 | [.02, .06] | .00 | [-.02, .02] |
| CA→NA→SRL | .00 | [.00, .01] | .00 | [.00, .01] |
| SS→NA→SRL | .01 | [.00, .02] | .01 | [.01, .02] |
| ECA→NA→SRL | .00 | [.00, .01] | .00 | [.00, .00] |
| ECA→CA→SRL | .00 | [-.01, .01] | .00 | [-.01, .01] |
| ECA→SS→SRL | .06 | [.03, .09] | .06 | [.03, .09] |
| CA→PA→SM | -.01 | [-.03, .00] | -.01 | [-.02, .00] |
| SS→PA→SM | .09 | [.04, .13] | .09 | [.04, .14] |
| ECA→PA→SM | .02 | [.01, .04] | .00 | [-.01, .01] |
| CA→NA→SM | .01 | [.00, .02] | .01 | [.00, .02] |
| SS→NA→SM | .02 | [.00, .03] | .02 | [.00, .03] |
| ECA→NA→SM | .00 | [.00, .01] | .00 | [-.01, .00] |
| ECA→CA→SM | .01 | [-.01, .02] | .01 | [-.00, .02] |
| ECA→SS→SM | .06 | [.03, .08] | .06 | [.03, .09] |
| ECA→CA→PA | -.02 | [-.03, .00] | -.01 | [-.03, .00] |
| ECA→SS→PA | .09 | [.05, .13] | .09 | [.05, .14] |
| ECA→CA→NA | -.01 | [-.03, .00] | -.01 | [-.03, .00] |
| ECA→SS→NA | -.03 | [-.05, -.01] | -.03 | [-.05, -.01] |

Note. AA = Academic achievement; LS = Life satisfaction; SRL = Self-regulated learning; SM = Scholastic motivation; PA = Positive emotions; NA = Negative emotions; CA = Cognitive abilities; SS = Soft skills; ECA = Extracurricular activities

**Supplementary figures**

**Figure S1** Prior (dotted curve), likelihood (continuous line curve) and posterior (colored curve) distributions of the effects of model 2. The thick horizontal line represents higher posterior density intervals.


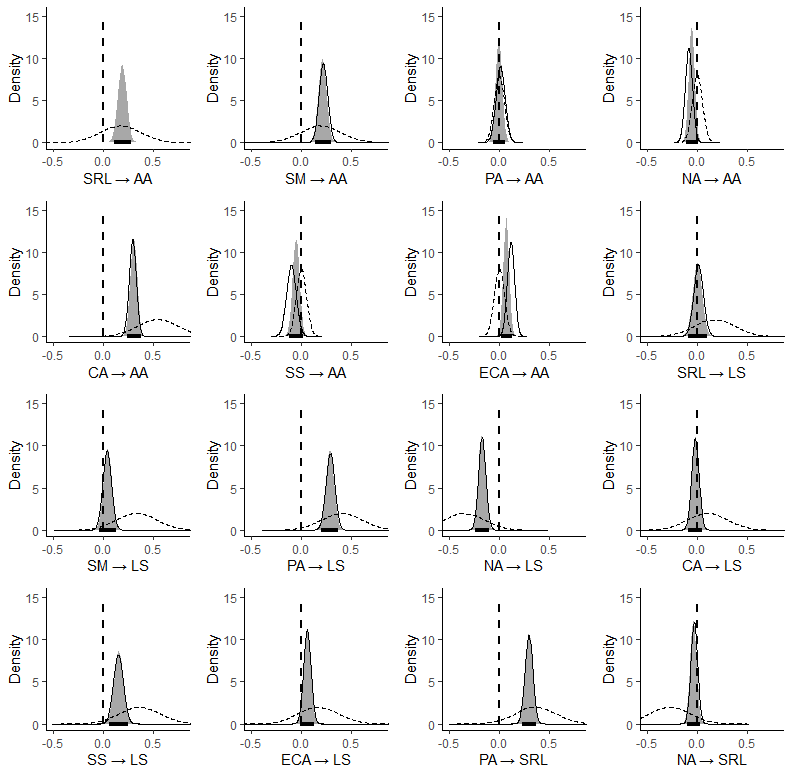


Note. AA = Academic achievement; LS = Life satisfaction; SRL = Self-regulated learning; SM = Scholastic motivation; PA = Positive emotions; NA = Negative emotions; CA = Cognitive abilities; SS = Soft skills; ECA = Extracurricular activities
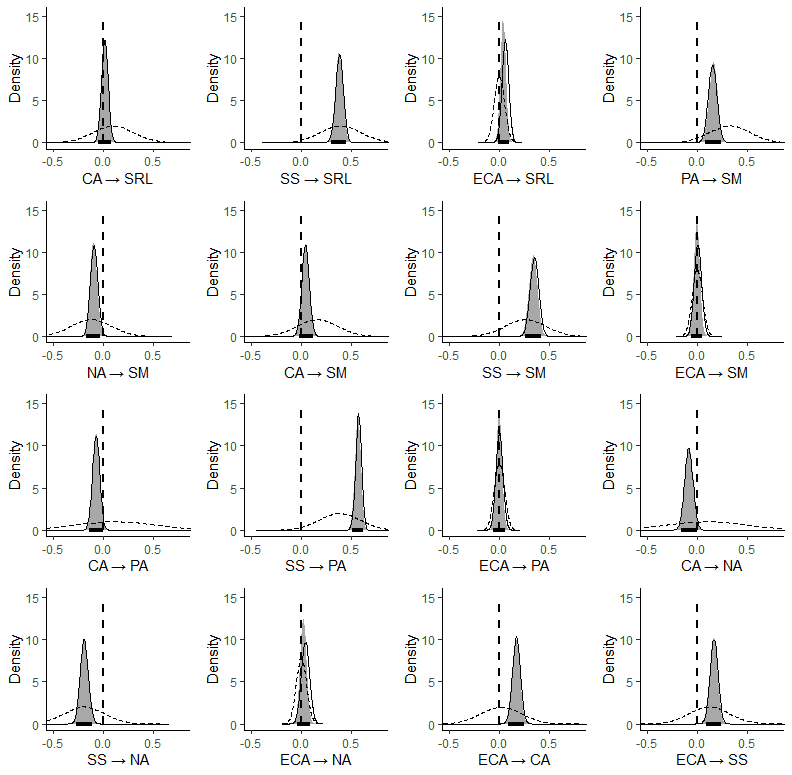


Note. AA = Academic achievement; LS = Life satisfaction; SRL = Self-regulated learning; SM = Scholastic motivation; PA = Positive emotions; NA = Negative emotions; CA = Cognitive abilities; SS = Soft skills; ECA = Extracurricular activities

**Figure S2.**

Single soft skills beta estimates and associated standard errors and HPDI (in grey)

**
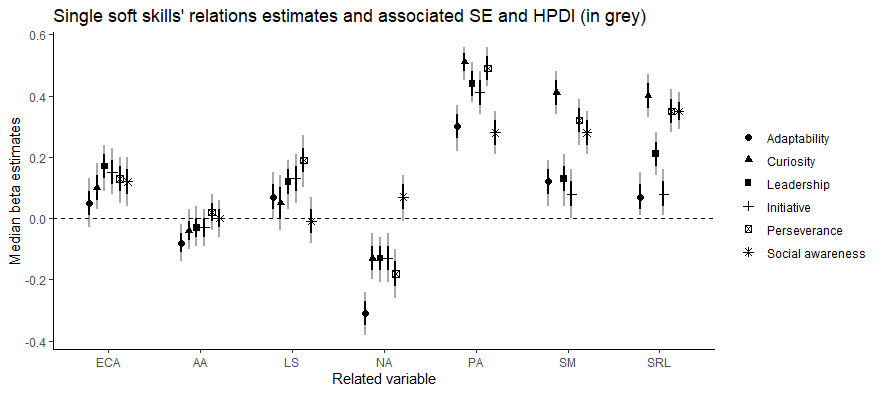
**

Note. ECA = Extracurricular activities; AA = Academic achievement; LS = Life satisfaction; NA = Negative emotions; PA = Positive emotions; SM = Scholastic motivation; SRL = Self-regulated learning
